# Exposure characteristics and cumulative risk assessment of bisphenol A and its substitutes: the Taiwan environmental survey for toxicants 2013

**DOI:** 10.3389/fpubh.2024.1396147

**Published:** 2024-05-23

**Authors:** Yu-Jung Lin, Hsin-Chang Chen, Jung-Wei Chang, Han-Bin Huang, Wan-Ting Chang, Po-Chin Huang

**Affiliations:** ^1^National Institute of Environmental Health Sciences, National Health Research Institutes, Miaoli, Taiwan; ^2^Department of Chemistry, Tunghai University, Taichung, Taiwan; ^3^Institute of Environmental and Occupational Health Sciences, School of Medicine, National Yang Ming Chiao Tung University, Taipei, Taiwan; ^4^School of Public Health, National Defense Medical Center, Taipei, Taiwan; ^5^Department of Medical Research, China Medical University Hospital, China Medical University, Taichung, Taiwan; ^6^Research Center for Precision Environmental Medicine, Kaohsiung Medical University, Kaohsiung, Taiwan; ^7^Department of Safety, Health and Environmental Engineering, National United University, Miaoli, Taiwan

**Keywords:** Taiwanese, endocrine disruptor, cumulative risk assessment, environmental pollutants, human health

## Abstract

**Introduction:**

Ever since the use of bisphenol A (BPA) has been restricted, concerns have been raised regarding the use of its substitutes, such as bisphenol S (BPS) and bisphenol F (BPF). Meanwhile, the EU European Food Safety Authority (EFSA) issued the new tolerable daily intake (TDI) after the latest re-risk assessment for BPA, which enforced the need for cumulative risk assessment in the population. This study was conducted to identify BPA and its substitute’s exposure characteristics of the general Taiwanese population and estimate the cumulative risk of bisphenol exposure.

**Methods:**

Urine samples (*N* = 366 [adult, 271; minor, 95]) were collected from individuals who participated in the Taiwan Environmental Survey for Toxicants 2013. The samples were analyzed for BPA, BPS, and BPF through ultraperformance liquid chromatography–tandem mass spectrometry. Daily intake (DI) levels were calculated for each bisphenol. Hazard quotients (HQs) were calculated with the consideration of tolerable DI and a reference dose. Additionally, hazard index (HI; sum of HQs for each bisphenol) values were calculated.

**Results:**

Our study found that the median level of BPA was significantly higher in adults (9.63 μg/g creatinine) than in minors (6.63 μg/g creatinine) (*p* < 0.001). The DI of BPS was higher in female (0.69 ng/kg/day) than in male (0.49 ng/kg/day); however, the DIs of BPF and BPS were higher in boys (1.15 and 0.26 ng/kg/day, respectively) than in girls (0.57 and 0.20 ng/kg/day, respectively). Most HI values exceeded 1 (99% of the participants) after EFSA re-establish the TDI of BPA.

**Discussion:**

Our study revealed that the exposure profiles and risk of BPA and its substitute in Taiwanese varied by age and sex. Additionally, the exposure risk of BPA was deemed unacceptable in Taiwan according to new EFSA regulations, and food contamination could be the possible source of exposure. We suggest that the risk of exposure to BPA and its substitutes in most human biomonitoring studies should be reassessed based on new scientific evidence.

## 1 Introduction

Bisphenol A (BPA), produced in large quantities worldwide, is a well-known endocrine-disrupting chemical (1). It is a synthetic monomer used in the high production of polycarbonate plastics and epoxy resins (2), with applications in various industries (3). These include thermal paper, toys, tableware, medical devices, polycarbonate bottles, food packaging, cosmetics, and personal care products (PCPs) (2, 4–7). Notably, BPA-containing products release BPA at room temperature, and the degree of BPA release is higher at higher temperatures, potentially resulting in higher risks of BPA exposure and associated health problems (8, 9). Although BPA exposure can occur through inhalation, dermal absorption, and non-dietary ingestion (10–12), the dietary ingestion of contaminated food is the main route of BPA exposure (13, 14). A growing body of evidence suggests that because of its estrogenic and anti-androgenic properties by disrupting normal signaling pathways for the endocrine system (15), exposure to BPA has been related to most diseases and its implications for public health are extensive (16–19). Briefly, BPA can cause substantial damage to tissues and systems, such as reproductive, immune, and neuroendocrine systems (7, 20–24) and also related to an increase in hormone-dependent pathologies, obesity, or type 2 diabetes (17).

Considering the negative effects of BPA on human health, multiple government bodies have implemented relevant regulations to reduce the level of BPA exposure, including banning BPA in food contact materials and packaging with bans on use in products used by infants (25, 26). Consequently, many chemical compounds with a chemical structure similar to that of BPA, such as bisphenol F (BPF) (27) and bisphenol S (BPS) (28), have emerged as BPA substitutes for use in consumer products (3, 29). Between 2012 and 2017, urinary BPA concentrations in Japanese children decreased on average by 6.5% per year (30). Otherwise, there was a significant decreasing trend in BPA concentrations in the Canadian population between 2007 and 2019 (31). However, some other substitutes of BPA have been regularly detected in human urine samples, including BPF and BPS (32). BPF has been the most important risk driver since 2000 in Japan (33), whereas BPS concentrations increased every year in Australia from 2012 to 2017 (34), suggesting that BPF and BPS have become a BPA replacement. Notably, most animal studies have reported that BPA and its substitutes (e.g., BPF and BPS) disturb the reproductive neuroendocrine system (35), increasing the risk of endocrine disruption (36). Moreover, BPF and BPS not only exhibit estrogenic and anti-androgenic properties but also interfere with glucocorticoid receptor signaling, which is similar to those of BPA (1, 20, 37), and this finding also supports the hypothesis that BPS poses a risk to human reproduction (38). Furthermore, BPS has also been classified as toxic to reproduction by the Risk Assessment Committee (RAC) of the European Chemicals Agency (ECHA) (Repr. 1B). In fact, the negative effects of BPS may be stronger than those of BPA (39). Although most studies have confirmed the health hazards of BPF and BPS (40–43), stringent regulations are yet to be implemented in multiple countries, including Taiwan.

Furthermore, the safety levels of BPA for humans established by different risk assessment authorities show disparities with respect to the range of exposure, which were carried out by regulatory agencies for the purpose of evaluation of the margin of safety (MOS) or proposal for a tolerable daily intake (TDI) (44). If cumulative intake above the safety level is expressed by a Health-based Guidance Value (HbGV), the TDI may be considered harmful to human health (45). In April 2023, the European Food Safety Authority (EFSA) re-evaluated the risks to public health from BPA in foodstuffs and established a TDI of 0.2 ng/kg body weight daily (46). Essentially, this change would ban BPA from food contact materials and most plastics used in consumer products in Europe. Therefore, further epidemiological studies and a re-evaluation of the association between potential health effects and realistic BPA exposure levels are warranted, especially in BPA substitutes.

However, in Taiwan, the Food and Drug Administration only banned the use of BPA in baby bottles in 2013, and there are no management regulations for BPA substitutes. Based on the new regulations by EFSA, it is still unknown whether Taiwanese populations are safe to be exposed to BPA, and there is still a lack of risk assessment of substitutes of BPA. Moreover, even though previous studies have pointed out that BPS and BPF are not safe substitutes for BPA (47), Taiwan has not yet conducted a study on these substances. Briefly, considering the impact of BPA and its substitutes on human health and its broader public health implications is essential (17). Hence, we analyzed urinary samples obtained from the general Taiwanese population to back-calculate daily intake (DI) levels of BPA and its substitutes, especially BPF and BPS. The objectives of this study were to (1) compare the concentration levels of BPA and its substitutes with other countries; (2) estimate the DIs of BPA and its substitutes using individual urinary levels, identifying bisphenol exposure characteristics, and (3) conduct the cumulative risk assessment of bisphenols.

## 2 Materials and methods

### 2.1 Ethics statement

This study was approved by the Research Ethics Committee of the National Health Research Institutes, Taiwan (EC1020206). Written informed consent was obtained from all participants before data collection.

### 2.2 Study participants and sampling

This study analyzed individuals who participated in the Taiwan Environmental Survey for Toxicants (TEST) 2013. The recruitment process has been published previously (48–52). Between May and December 2013, we selected 17 townships from 11 Taiwanese cities and counties and 1 remote island region (Penghu County). First morning spot urine was collected; to collect demographic data (e.g., anthropometry index, socioeconomic status, smoking habits, and residence), an extensive questionnaire was administered.

### 2.3 Analytical method for detecting bisphenol

We previously described the processes of method optimization, development, and validation for the analysis of bisphenol in urine (53). Bisphenol standards were of analytical grade and accompanied by a certificate of analysis (at minimum). The target analytes BPA BPS and BPF were acquired from AccuStandard (New Haven, CT, USA) and Toronto Research Chemicals (LGC, Manchester, NH, USA), respectively. The stable isotopically labeled internal standards (SIL-ISTDs) of 100 μg/mL ^13^C_12_-BPA was obtained from Cambridge Isotope Laboratories (Tewksbury, MA, U.S.A.); BPS-d_8,_ and BPF-d_10_ were purchased from Toronto Research Chemicals (LGC, Manchester, NH, U.S.A.). For neat standards, standards in stock solutions, working solutions of the bisphenols, and SIL-ISTDs were diluted to appropriate concentrations with MeOH. Our previous study (53) described these reagents in detail.

An online system was used to interpret the results of ultraperformance liquid chromatography–tandem mass spectrometry. In brief, mid-stream urine samples were collected in the morning and stored at −80°C; before analysis, the samples were thawed at 4°C for 24 h. The urine (100 μl) was mixed with 20 μl of methanol containing stable isotope-labeled internal standards, 5 μl of β-glucuronidase, and 20 μl of 1.0 M of ammonium acetate (aqueous) and then vigorously shaken on a Vortex-2 Genie Shaker (Scientific Industries, USA). The sample was incubated at 40°C for 1 h after rotation, and 135 μl of 0.1% formic acid (aq) were added and mixed for extraction by supported liquid extraction (SLE). In the final step, the analytes were eluted with 100 μl of MeOH and 100 μl of Milli-Q water and were ready for injection.

The recovery and matrix effect of supported liquid extraction (SLE) and the linearity of the isotope dilution calibration curves have been validated. The limit of detection was 0.1 (ng/ml) for BPA, BPF, and BPS. Quality control samples were prepared using the same protocol for the calibrators, except for three spiked concentrations. To assess within-run and between-run assay variability, the spiked samples were analyzed after the analysis of every 10 samples. On the basis of the quality control criteria set by the European Medicines Agency, the test accuracy and precision were estimated to be more than 85 and 15%, respectively (54). In brief, the requirements for limits of detection, lower limits of quantification, and within-run and between-run accuracy and precision have also been achieved and shown in Supplementary Tables S1–S3.

### 2.4 DI calculation of BPA and its substitutes

We estimated the DI of each bisphenol for multiple age groups (adults and minors). To calculate DIs, we analyzed data regarding urinary bisphenol levels and used a back-calculation method (50, 51, 55). Equation 1 presents the formula used for back-calculation (55). The urinary excretion fraction (F_UE_) was set to a value of 1 based on relevant studies (56–59); approximately 100% of BPA and its substitutes (BPS and BPF) were excreted through urine within 24 h.


(1) $$\begin{array}{l} \mathrm{Daily}\;\mathrm{intake}\;\mathrm{of}\;\mathrm{bisphenol}\;\left[\frac{\left(\frac{\mathrm{\mu g}}{\mathrm{kg}}\right)}{\mathrm{day}}\right]\\ =\frac{UE\;\left(\frac{\mathrm{\mu g}}{g}\right)\times CE\;\left(\frac{\frac{\mathrm{mg}}{\mathrm{kg}}}{\mathrm{day}}\right)}{F_{UE}\times BW\;\left(Kg\right)\times 1000\left(\frac{\mathrm{mg}}{g}\right)}\; \end{array}$$

where UE denotes the level of creatinine-adjusted bisphenol (microgram of bisphenol per gram of creatinine), BW denotes body weight, and CE denotes the level of daily creatinine excretion (60, 61), which is calculated as follows:

For adults (age ≥ 18 years):

CE = 1.93 × (140 − age) × body weight^1.5^ × height^0.5^ × 10^−3^ (for male).

CE = 1.64 × (140 − age) × body weight^1.5^ × height^0.5^ × 10^−3^ (for female).

For minors (age ≥ 3 to <18 years):

CE = height × {6.265 + 0.0564 × (height − 168)} (for boys with a height of <168 cm).

CE = height × {6.265 + 02550 × (height − 168)} (for boys with a height of ≥168 cm).

CE = 2.045 × height × exp.{0.01552 × (height − 90)} (for girls).

### 2.5 Cumulative risk assessment of BPA and its substitutes

For cumulative risk assessment, we calculated hazard quotients (HQs) and HI values. HQs were calculated to quantitatively assess the potential health hazards of bisphenol. HI is the sum of HQs for each bisphenol. An HI value of >1 suggests adverse health effects (62). HQ was derived using the corresponding TDIs or reference doses (RfDs). The RfDs of BPA and BPS were identified from a relevant study (63, 64), and these values were 12,500 and 13,700 (ng/kg/day), respectively, and were determined on the basis of their anti-androgenic effects on animal models (Reference Doses for Anti-Androgenicity, RfD AA). However, the RfD AA of BPF remains not to be established. We assume that the RfD AA of BPF was obtained by converting to the same molar levels used for BPA, and the value was 11,000 (ng/kg/day) (Eq. 2).

The European Food Safety Authority (65) recommends a TDI of 4,000 ng/kg of body weight/day for BPA. Lin et al. (66) recommended a TDI of 4,000 (ng/kg/day) for BPF, and Mok et al. (64) recommended a TDI of 4,400 (ng/kg/day) for BPS (Eq. 3). In 2023, EFSA re-established a new TDI of 0.2 ng/kg body weight per day for BPA, which is 20,000 times lower than the previous TDI of 4,000 ng/kg of body weight/day (46). However, the TDI of BPF or BPS remains to be established, in which case we assume that they have the same TDI as BPA (Eq. 4).

The following formula was used to calculate HQs (67):


(2) $${HQ}_{RfD\;\mathrm{AA}}=\frac{DI}{\mathrm{RfDs}\;}$$

(3) $${HQ}_{TDI\;for\;\mathrm{EFSA}\;2015}=\frac{DI}{TDI}$$

(4) $${HQ}_{TDI\;for\;\mathrm{EFSA}\;2023}=\frac{DI}{TDI}$$

The following formula was used to calculate HI values:

Scenario 1 and 2 [HQ_TDI_ Based on (46, 65)]: HI_BPs_ = HQ_BPA TDI_+ HQ_BPF TDI_ + HQ_BPS TDI_.

Scenario 3 HI_BPs_ = HQ_BPA RfDAA_ + HQ_BPS RfDAA_.

Scenario 4 HI_BPs_ = HQ_BPA RfDAA_ + HQ_BPF RfDAA_ + HQ_BPS RfDAA_.

### 2.6 Statistical analysis

The participants were divided into two age groups: adults and minors. We also divided participants into five psychology age groups as abovementioned. Descriptive statistics for participant demographics are presented in terms of medians and interquartile ranges for continuous variables and as numbers and percentages for categorical variables. The distribution of urinary bisphenol levels is presented in terms of geometric means, minima, maxima, and percentiles (25th, 50th, and 75th) for the two age groups. The comparison among groups was analyzed by performing non-parametric tests (Mann–Whitney *U* Test or Kruskal–Wallis Test). Additionally, the comparison among groups was analyzed by performing parametric tests (chi-square test or *t*-test) in demographic data. The total number of types of personal care products (PCPs) used by the participants was calculated. The use of the following four product types was assessed: body wash, lotion, perfume, and nail polish. The cumulative number of uses of PCPs was calculated as the sum of usage of products including body wash, lotion, perfume, and nail polish. The use of ≥2 types of PCPs, a single type of PCP and no use PCP, indicated high and low usage levels, respectively. To assess PCP use by the different age groups, the Mann–Whitney U and Kruskal–Wallis tests were performed. The summary metric for BPs (ΣBPs) was calculated by summarizing the molar concentrations of the measured BPs (33, 64, 68). All bisphenol measurements, including the molar sum, were divided by urinary creatinine to adjust for urine dilution. All statistical analyses were performed using SAS (version 9.4; SAS Institute, Cary, NC, USA). A *p*-value of <0.05 was considered significant.

## 3 Results

### 3.1 Demographic characteristics and exposure profiles of the study population

A total of 394 TEST participants were included in this study. We excluded 28 individuals who lacked urinary bisphenol data. Finally, 366 individuals (adults [age, ≥18 years], 271; minors [age, 7 to 18 years], 95) were included in the analysis. The general and sociodemographic characteristics of the participants stratified by age are shown in Table 1. For adults, the most prevalent demographic characteristics were female sex (52.8%), age of 40–65 years (46.9%), living in Northern Taiwan (31%), married (72.7%), received a college- or graduate-level education (35.1%), non-smoker (75.9%), and no reported pesticide use at home (76.0%). Furthermore, most of the participants did not consume alcohol (86.9%) or chew betel nut (93.4%). Just over half of the minors were boys (57.9%), 51.6% of them aged 7–12 years, and 52.1% were passive smokers.

**Table 1:** Demographic characteristics of all participants in this study (*N* = 366).

Characteristics	Item	Minors (<18 years, *n* = 95)		Adults (≥18 years, *n* = 271)	
		*n*	%	*n*	%
Gender	Girl/Female	40	42.1	143	52.8
	Boy/Male	55	57.9	128	47.2
Age (years)	7–12/18–40	49	51.6	64	23.6
	12–18/40–65	46	48.4	127	46.9
	65 and older	–	–	80	29.5
Region	Northern Taiwan	31	32.6	84	31.0
	Central Taiwan	15	15.8	37	13.7
	Southern Taiwan	22	23.2	77	28.4
	Eastern Taiwan	12	12.6	46	17.0
	Remote island	15	15.8	27	10.0
Marriage status	Single	94	99.0	46	17.0
	Married	1	1.0	197	72.7
	Divorce/widowed	0	0	28	10.3
Education	≦Elementary school	49	51.6	74	27.3
	Junior high school	29	30.5	39	14.4
	Senior high school	17	17.9	63	23.2
	≧College/graduates	0	0	95	35.1
Annual family income (USD)^a^	Below 15,625	37	42.1	151	58.1
	More than 15,625	51	57.9	109	41.9
Cigarette smoking^b^	Yes/No	2/93	2.1/97.9	65/205	24.1/75.9
Passive smoker^c^	Yes/No	49/45	52.1/47.9	135/135	50.0/50.0
Incense sticks^d^	Yes/No	29/66	30.5/69.5	147/123	54.4/45.6
PCPs usage^e^	Yes/No	83/11	88.3/11.7	197/69	74.1/25.9
Alcohol consumption^f^	Yes/No	1/93	1.1/98.9	35/232	13.1/86.9
Tea drinking^g^	Yes/No	46/49	48.4/51.6	156/114	57.8/42.2
Coffee drinking^g^	Yes/No	6/89	6.3/93.7	114/157	42.1/57.9
Betel nut chewing^h^	Yes/No	1/94	1.1/98.9	18/253	6.6/93.4
Pesticide use at home^i^	Yes/No	26/69	27.4/72.6	65/206	24.0/76.0

^a^The currency exchange rate of converting USD to the new Taiwan dollar is 1:32. ^b^Subjects who self-reported consuming at least one cigarette per day. ^c^Subject who self-reported as a lifelong non-smoker (never smoked) but involuntary inhalation of smoke from cigarettes or other tobacco. ^d^Subject who self-reported as having burnt incense at home ≥ weekly basis over the past 5 years. ^e^Subject who self-reported using at least one kind of PCPs, including body wash, lotion, perfume, and nail polish. ^f^Subject consuming at least one bottle of alcoholic drink per week. ^g^Subjects consuming at least one cup of tea or coffee per week. ^h^Subject chewing at least one betel nut per week. ^i^Subject who self-reported using household pesticides to control pests.

**Figure 1:**
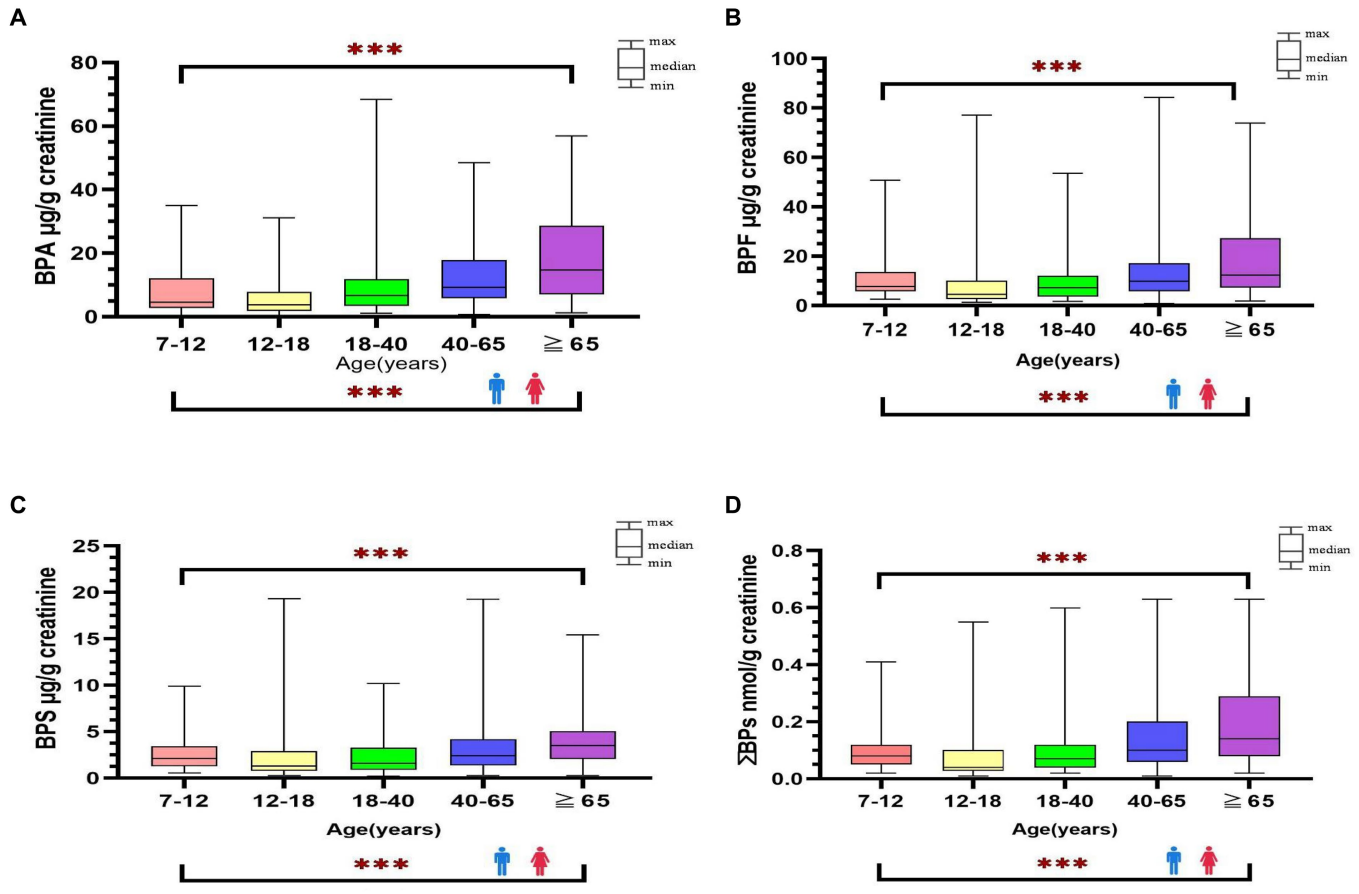
Distribution of bisphenol A and its substitutes [BPA **(A)**, BPF **(B)**, BPS **(C)**, ΣΒΡs **(D)**] in general Taiwanese (*N* = 366) by age and sex groups. ***: *p*-value <0.001 between different age groups.

The detection rate of BPA and its substitutes was 100% in all urine samples. Table 2 presents the distribution of urine creatinine-adjusted BPA, BPF, BPS, and ΣBPs in the participants stratified by age, sex, and location (Supplementary Table S4 presents the urine creatinine-unadjusted results). The median level of BPA and its substitutes was significantly higher in adults than in minors (BPA: 9.45 vs. 4.08; BPF: 9.63 vs. 6.63; BPS: 2.43 vs. 1.67 μg/g creatinine; ΣBPs: 0.10 vs. 0.06 nmol/g creatinine; *p* < 0.001). Among adults, the urinary levels of BPA, BPF, and BPS were slightly higher in women than in men (BPA: 11.07 vs. 8.18; BPF: 11.2 vs. 8.55; BPS: 3.05 vs. 2.11 μg/g creatinine; ΣBPs: 0.11 vs. 0.09 nmol/g creatinine.; *p* < 0.01); same as minors, the levels were higher in girls than in boys (BPA: 12.77 vs. 5.59; BPF: 7.24 vs. 4.88; BPS: 2.58 vs. 1.36; ΣBPs: 0.09 vs. 0.05 nmol/g creatinine; *p* < 0.01). The participants were further stratified by location; thus, five region-based groups were formed. However, no significant difference in bisphenol levels was observed in five regions, and it showed that bisphenol levels were similar in each region of Taiwan. The median levels of bisphenol (creatinine-adjusted) in all participants were significantly increased along with increasing age (*p* < 0.001, Figure 1).

**Table 2:** Distribution of bisphenol concentration (μg/g Cr) in the general Taiwanese population (*N* = 366).

Characteristics	**BPA**							**BPF**							**BPS**							**ΣBisphenols** ^**a**^						
	GM	Min	Selected percentiles			Max	*p-*value ^b^	GM	Min	Selected percentiles			Max	*p-*value ^b^	GM	Min	Selected percentiles			Max	*p-*value^b^	GM	Min	Selected percentiles			Max	*p-*value ^b^
			25th	50th	75th					25th	50th	75th					25th	50th	75th					25th	50th	75th		
All sample	7.84	0.30	3.99	8.18	16.12	68.44		8.98	0.88	4.89	8.66	15.95	84.33		2.18	0.20	1.22	2.24	4.07	19.33		0.09	0.01	0.05	0.09	0.17	0.63	
Minors	4.17	0.30	2.68	4.08	11.27	35.00	<0.001	6.85	1.25	3.60	6.63	12.04	77.00	<0.001	1.73	0.30	1.00	1.67	3.18	19.33	<0.001	0.06	0.01	0.03	0.06	0.11	0.55	<0.001
Adults	9.79	0.71	5.73	9.45	18.26	68.44		9.87	0.88	5.58	9.63	17.84	84.33		2.36	0.20	1.34	2.43	4.35	19.28		0.11	0.01	0.06	0.10	0.20	0.63	
Gender							<0.001							<0.001							<0.001							<0.001
Female	9.56	0.30	5.46	10.28	18.41	68.44		10.49	0.88	6.11	10.03	19.31	77.00		2.59	0.28	1.51	2.75	4.42	19.33		0.11	0.01	0.06	0.11	0.20	0.63	
Male	6.44	0.36	3.42	6.51	12.78	50.21		7.68	1.28	3.73	7.64	13.98	84.33		1.83	0.20	1.07	1.69	3.34	19.28		0.08	0.01	0.04	0.08	0.15	0.63	
Regional area							0.78							0.673							0.803							0.663
Northern	8.58	0.36	4.77	9.42	15.48	49.49		9.50	1.79	5.45	8.80	15.90	64.68		2.30	0.30	1.25	2.45	4.08	15.11		0.10	0.01	0.06	0.10	0.17	0.57	
Central	7.11	0.30	3.10	8.39	17.18	68.44		8.29	1.25	3.58	7.62	15.22	77.00		2.03	0.20	0.88	2.16	4.44	19.33		0.09	0.01	0.04	0.09	0.19	0.60	
Southern	7.55	0.41	4.00	6.89	15.89	56.98		8.30	1.28	4.88	7.85	14.99	64.61		2.13	0.22	1.21	2.13	3.59	15.43		0.09	0.01	0.05	0.08	0.16	0.63	
Eastern	7.75	0.42	3.84	7.28	16.56	50.21		9.99	1.63	4.83	9.22	24.39	84.33		2.38	0.36	1.30	2.45	4.52	19.28		0.10	0.02	0.06	0.09	0.20	0.63	
Remote island	7.70	0.71	4.08	8.19	17.01	28.79		8.82	0.88	4.15	9.87	16.33	50.69		1.90	0.28	1.29	2.03	3.36	9.90		0.09	0.01	0.04	0.11	0.16	0.41	
**Adults**																												
Gender							0.007							0.008							0.004							0.005
Female	11.1	0.71	6.51	11.07	20.13	68.44		11.16	0.88	6.26	11.20	21.51	64.61		2.71	0.28	1.56	3.05	4.85	15.43		0.12	0.01	0.07	0.11	0.23	0.63	
Male	8.51	1.03	4.60	8.18	15.89	50.21		8.60	1.63	4.55	8.55	14.52	84.33		2.02	0.20	1.22	2.11	3.51	19.28		0.09	0.02	0.05	0.09	0.15	0.63	
Age (years)							<0.001							<0.001							<0.001							<0.001
18–40	6.53	1.03	3.43	6.73	11.72	68.44		6.96	1.63	3.72	7.12	11.94	53.52		1.66	0.20	0.89	1.58	3.28	10.19		0.07	0.02	0.04	0.07	0.12	0.60	
40–65	9.67	0.71	5.89	9.26	17.87	48.53		9.89	0.88	5.69	9.77	17.10	84.33		2.32	0.28	1.34	2.40	4.20	19.28		0.10	0.01	0.06	0.10	0.20	0.63	
65 and older	13.82	1.25	7.13	14.78	28.52	56.98		13.02	1.89	7.29	12.35	27.24	73.92		3.22	0.30	2.08	3.51	5.03	15.43		0.14	0.02	0.08	0.14	0.29	0.63	
**Minors**																												
Gender							0.007							0.02							0.008							0.005
Girl	5.59	3.24	6.55	12.77	35.00	0.30		8.40	1.25	6.00	7.24	12.39	77.00		2.21	0.33	1.30	2.58	3.31	19.33		0.08	0.01	0.05	0.09	0.12	0.55	
Boy	3.36	1.60	3.68	5.59	31.18	0.36		5.90	1.28	2.84	4.88	11.43	61.41		1.44	0.30	0.79	1.36	2.14	15.00		0.05	0.01	0.03	0.05	0.09	0.44	
Age (years)							0.439							0.003							0.024							0.014
7–12	4.37	0.36	3.02	4.59	12.01	35.00		8.59	2.48	5.94	7.64	13.37	50.69		2.05	0.56	1.28	2.14	3.26	9.90		0.08	0.02	0.05	0.08	0.12	0.41	
12–18	3.96	0.30	1.90	3.75	7.21	31.18		5.38	1.25	2.53	4.58	10.03	77.00		1.44	0.30	0.79	1.30	2.81	19.33		0.05	0.01	0.03	0.04	0.10	0.55	

^a^ΣBisphenols (the bisphenol weighted molar sum). ^b^Comparison of urine creatinine-adjusted bisphenol levels between adults and minors (e.g., sex) using the Mann–Whitney *U* test, above of two groups were using the Kruskal–Wallis test.

We found that 46.5% of the adult participants were high users of PCPs, whereas 53.5% were low users. Most of the minor participants (66.7%) were low users. Women (83.2%) self-reported the use of lotion more often than men (37.3%) (*p* < 0.001). Most participants self-reported eating fried or barbecued food at least once a month regardless of their age (fried vs. barbecued food: adults, 80.8% vs. 91.5%, respectively; minors, 90.6% vs. 82.3%, respectively; Supplementary Table S5). The median concentrations of bisphenols (creatinine-adjusted) in participants who used lotion were higher than in those with no use (BPA: 9.60 vs. 6.61 μg/g Cr; BPF: 10.36 vs. 7.02 μg/g Cr; BPS: 2.59 vs. 1.89 μg/g Cr; ΣBPs: 0.1 vs. 0.08 nmol/g Cr; *p* < 0.001), although the concentrations of BPA and its substitutes in participants with higher level of body wash use were lower than in those with no use. We also found that participants who reported long-term use of medication had significantly higher BPA and its substitutes (BPA: 11.27 vs. 6.72 μg/g Cr; BPF: 10.47 vs. 7.48 μg/g Cr; BPS: 3.11 vs. 1.93 μg/g Cr; ΣBPs: 0.11 vs. 0.08 nmol/g Cr; *p* < 0.001) (Supplementary Table S6). However, the median levels of BPA and its substitutes were not significant among different BMI weight status categories within each age group (Supplementary Table S7).

### 3.2 Estimated DIs of bisphenol A and its substitutes

**Figure 2:**
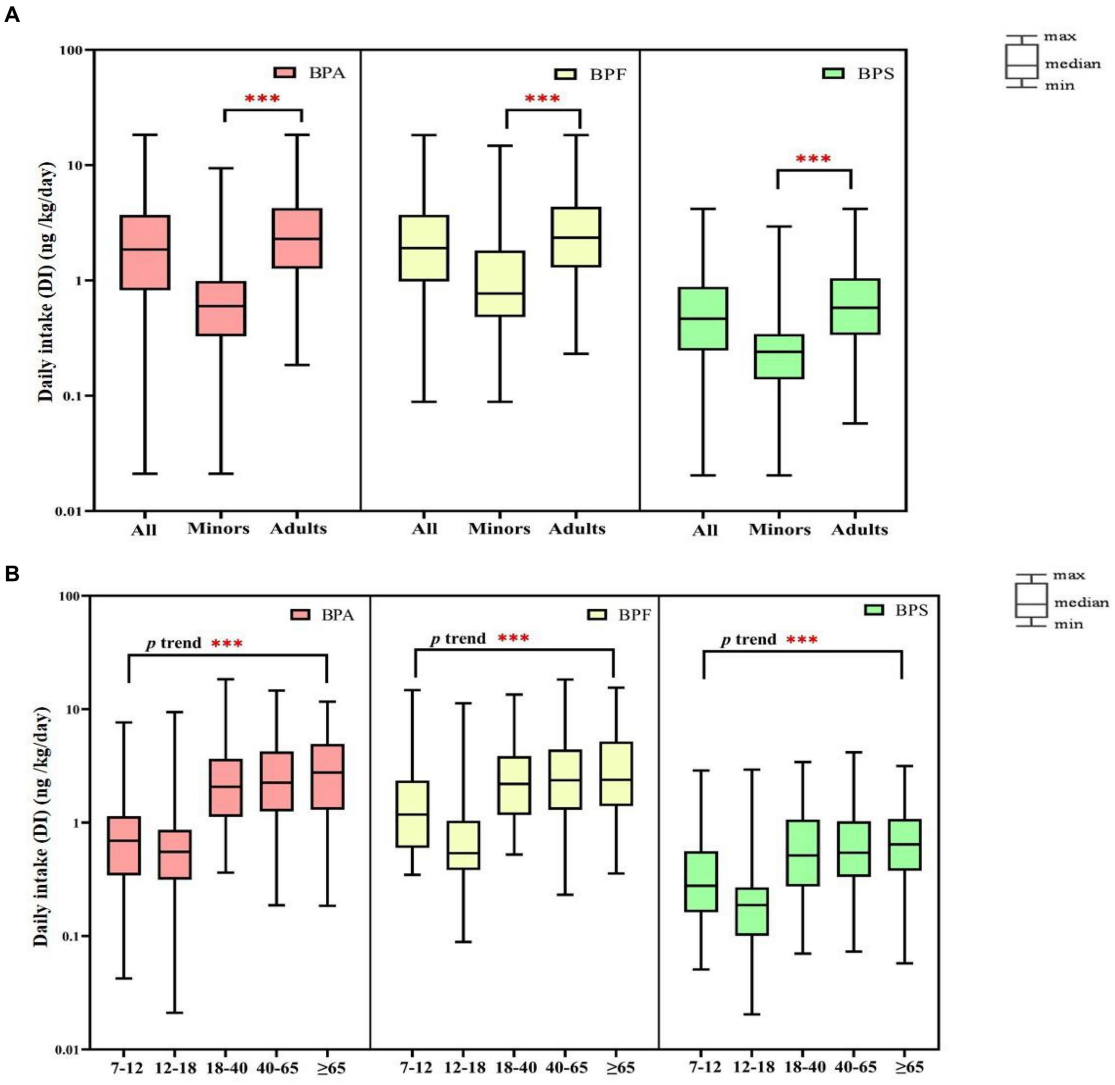
Daily intake (DI) of bisphenol A and its substitutes in all participants (*N* = 366) by age (**A**: minors and adults; **B**: different age). ***p-value <0.001 between different age groups.

We performed comprehensive estimated DI levels for BPA, BPF, and BPS; the results are presented in Figure 2 (Supplementary Tables S8, S9). The median DI values in all participants of BPA, BPF, and BPS were 1.86, 1.91, and 0.47 ng/kg/day, respectively. In adults, the median DIs of BPA, BPF, and BPS were 2.29, 2.35, and 0.58 ng/kg/day, respectively, and the median DI of BPS was significantly higher in women than in men (0.69 vs. 0.49 ng/kg/day, respectively, *p* = 0.032). In minors, the median DIs of BPA, BPF, and BPS were 0.60, 0.77, and 0.24 ng/kg/day, respectively, and the DIs of BPF and BPS were higher in boys than in girls (BPF: [1.15 vs. 0.57 ng/kg/day]; BPS: [0.26 vs. 0.20 ng/kg/day]). Overall, the median DI values of BPA and its substitutes were significantly lower in minors than in adults (*p* < 0.001). Furthermore, the median levels of the bisphenol DI in all participants were significantly increased along with increasing age (*p* < 0.001, Figure 2).

### 3.3 Estimated cumulative risk assessment of BPA and its substitutes

**Figure 3:**
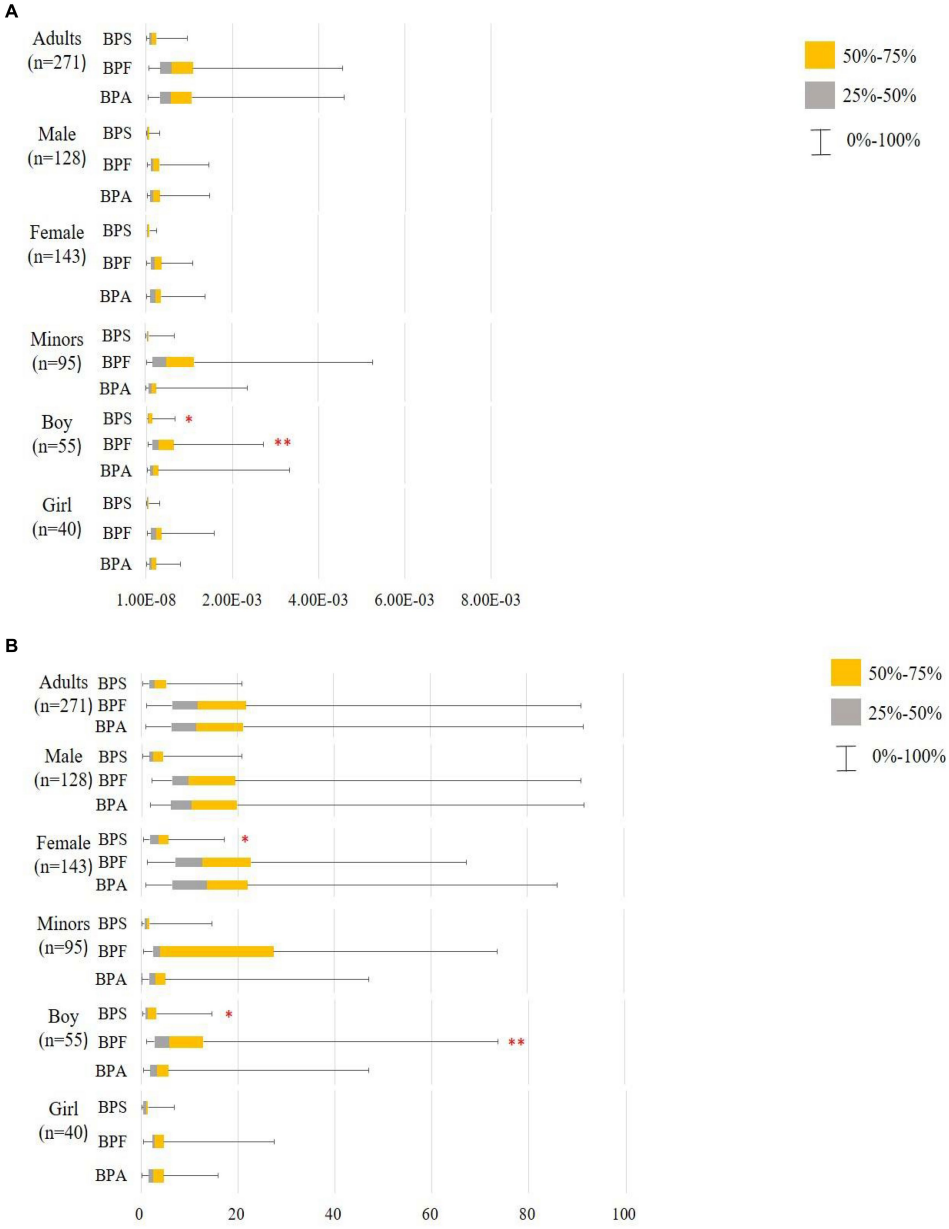
Hazard quotient (HQ) of bisphenol A and its substitutes in all participants (minors: n95 and adults: *n* = 271). **: *p* < 0.05; *p* < 0.05. **(A)** Scenario 1: Based on the TDI by EFSA (65) and thresholds derived by Mok et al. (64), Lin et al. (66) BPA and BPF TDI 4,000 ng/kg/day, BPS TDI 4,400 ng/kg/day. **(B)** Scenario 2: Based on the TDI by EFSA (46) and assumption that BPF and BPS have the same TDI; BΡΑ and its substitutes TDI 0.2 ng/kg/day; using the Mann–Whitney U test to compare HQTDI in different sex and labeled on the higher median value.

Using the RfD for anti-androgenicity (RfD AA) and TDI values, we calculated the HQs for adults and minors (Supplementary Tables S8, S9, Scenarios 1–4). Regardless of whether the HQs were calculated based on the TDI in Scenario 1 or the RfD AA in Scenario 3 and Scenario 4, the values were < 1 for BPA and its substitute. This indicates that the exposure levels were well below those where a substantial risk would be increased. Notably, EFSA (46) re-established the TDI for BPA (0.2 ng/kg/day), and we assumed that BPF and BPS had the same TDI (Scenario 2); the median of HQ_TDI_ for BPA and its substitutes exceeds 1 indicating health concerns, whether in adults (BPA: 11.45; BPF: 11.76; BPS: 2.90) or minors (BPA: 2.99; BPF: 3.85; BPS: 1.20). Among adults, the HQ_TDI_ of BPS was significantly higher in women than in men (3.45 vs. 2.43, respectively; *p* = 0.03). Additionally, the HQ_TDI_ of BPF and BPS was higher in boys than in girls (BPF, 5.75 vs. 2.83, respectively, *p* = 0.005; BPS, 1.28 vs. 1.02, respectively, *p* = 0.026). Overall, the HQ values were lower in minors than in adults (Figure 3).

**Figure 4:**
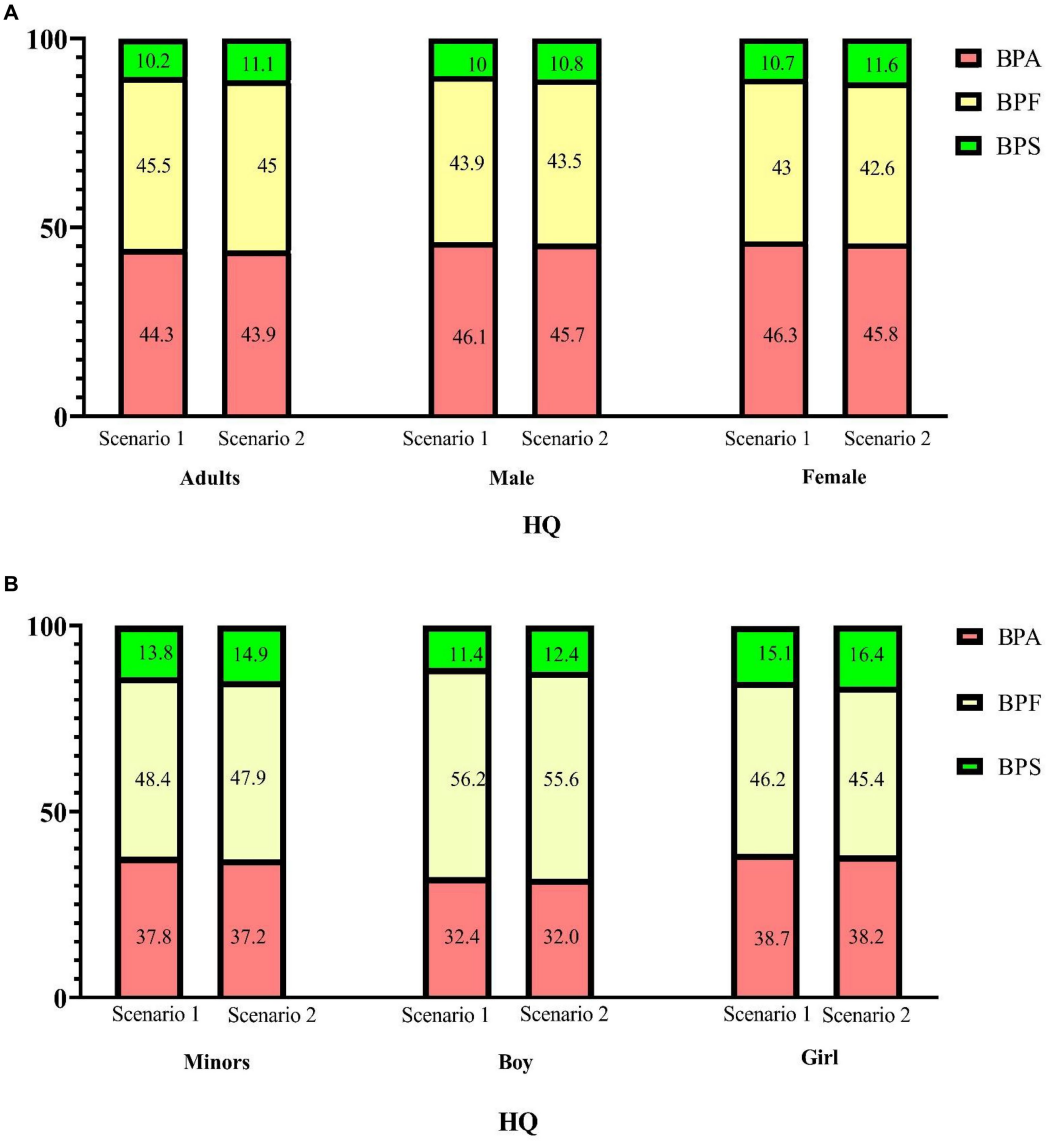
Percentage contribution of each hazard quotient (HQ) to the hazard index (HI) for bisphenol A and its substitutes. (**A**: adults; **B**: minors) Scenario 1: HQ-DI/TDI, BPA and BPF TDI 4,000 ng/kg/day, BPS TDI 4,400 ng/kg/day; Scenario 2: HQ-DI/TDI, BPA and its substitutes TDI = 0.2 ng/kg/day.

The median HI values (addition of the corresponding HQ_TDI_ values for BPA and its substitutes, Scenario 1 in Supplementary Tables S8, S9) were significantly higher in adults than in minors (*p* < 0.001). In adults, the median HI value was 1.29 × 10^−3^, the 95th percentile HI value was 4.55 × 10^−3^, and the maximum HI value was 8.39 × 10^−3^; BPF contributed to approximately 45.5% of the total health hazard (in terms of HI values; Figure 4). In minors, the median HI value was 4.10 × 10^−4^, the 95th percentile HI value was 2.23 × 10^−3^, and the maximum HI value was 6.25 × 10^−3^, of which 48.4% of the HI values were from the BPF of HQ contribution. In adults (regardless of sex), the contribution of BPA to the total health hazard was higher than that of BPF and BPS. By contrast, in minors (regardless of sex), the contribution of BPF to the total health hazard was higher than that of BPA and BPS (Table 3).

**Table 3:** International comparison of median levels among different studies for concentrations of BPA, BPF, and BPS from different countries (μg/L).

**Study, region**	**TESTs, Taiwan** ^**a**^					**NHANES, USA** ^b^					**CHMS, Canada** ^c^					**KoNEHS, Korea** ^d^	**GerES V, Germany** ^e^
Year	2013					2013–2014					2012–2013					2012–2014	2014–2017
Age	6–11 years	12-19 years	20–39 years	40–59 years	≧60 years	6–11 years	12–19 years	20–39 years	40–59 years	≧60 years	6–11 years	12–19 years	20–39 years	40–59 years	60–79 years	≧19 years	3–17 years
N	49	49	61	96	111	409	459	598	607	603	1,004	992	1,040	1,075	1,038	6,478	515
	Median	Median	Median	Median	Median	Median	Median	Median	Median	Median	Median	Median	Median	Median	Median	Median	Median
BPA	4.83	4.93	7.75	8.16	7.49	1.34	1.14	1.47	1.18	1.04	1.20	1.40	1.10	1.10	0.88	1.49	1.82
BPF	7.22	6.30	7.81	7.86	7.82	0.27	0.37	0.36	0.34	0.34	–^f^	–	–	–	–	–	–
BPS	1.75	1.97	2.11	1.96	1.92	0.27	0.30	0.43	0.35	0.32	–	–	–	–	–	–	–

^a^This study; ^b^NHANES, National Health and Nutrition Examination Survey 2013–2014 (32); ^c^CHMS, Canadian Health Measures Survey Cycle 3 (2012–2013) (69); ^d^KoNEHS, Korean National Environmental Health Survey (2012–2014) (70); ^e^GerES V, German Environmental Survey 2014–2017 (107); ^f^–data not reported.

**Figure 5:**
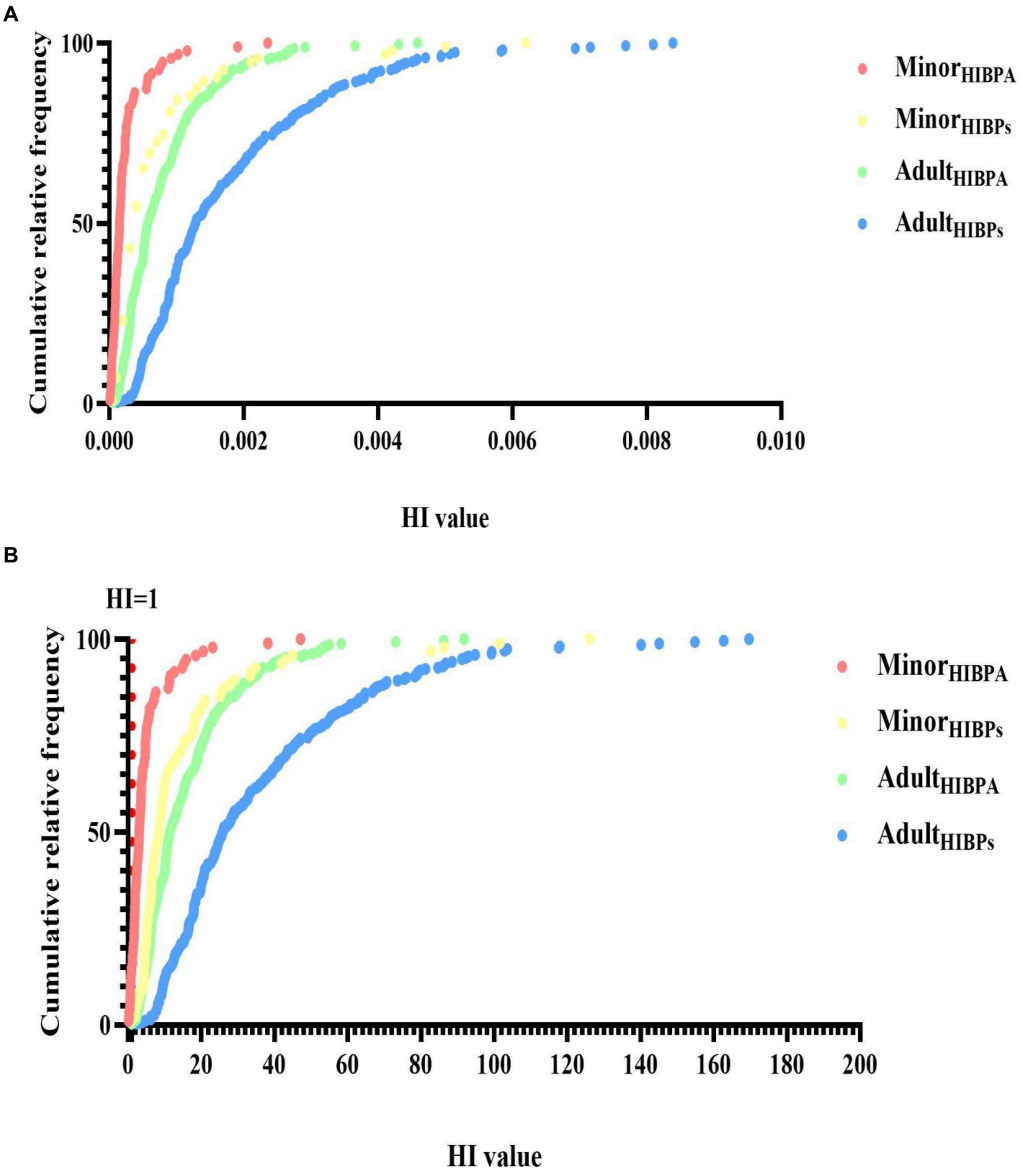
Cumulative relative frequency of bisphenol A and its substitutes’ hazard index (HI, SHQTDI) in all participants (minors: n95 and adults: *n* = 271) (**A**: [TDI based on EFSA (65)]; **B**: [TDI based on (46)]). The dotted line represents the border over which there is a substantial risk.

EFSA re-established the TDI of BPA to 0.2 ng/kg/day in Scenario 2. More than 95.6% of the 366 individuals in our study had an HI_BPA_ exceeding 1. When we assume that the TDI of BPF and BPS have the same TDI as BPA, more than 99% of the 366 individuals in our study had a cumulate HI_BPs_ value exceeding 1, indicating that bisphenol exposure risk was indeed a cause for concern. Consequently, as shown in Figure 5, most participants’ HI is higher than 1 when EFSA re-establishes TDI. Notably, 5% of adults were 92.23-fold higher than acceptable HI (<1). Adults had a higher HI than minors (25.88 vs. 8.30, respectively, *p* < 0.001), in which 45% of the HI values were from the BPF of HQ contribution (Figure 4). Moreover, the median of HI was significantly higher in women than in men (28.99 vs. 23.20, respectively, *p* = 0.045). By contrast, the median of HI was significantly higher in boys than in girls (9.44 vs. 6.99, respectively, *p* = 0.008).

The RfD of BPF remains to be established for its anti-androgenic effects (Scenario 3). When HQs were calculated using the RfD AA values, the median HI values (addition of the HQ_RfD_ values for BPA and BPS) were 2.27 × 10^−4^ in adults and 6.17 × 10^−5^ in minors. The contribution of BPA HQ to the HI value was approximately >80% in adults. Moreover, the percentage contribution of BPA (approximately 47%) to the HI value was similar to that of BPS HQ (approximately 50%) in minors. We assume that the RfD AA of BPF was obtained by conversion to the same molar levels used for BPA (RfD for BPF, 11,000 ng/kg/day), and the contribution of BPF HQ to the total health hazard was approximately 50% in adults and minors (Scenario 4).

### 3.4 Bisphenol levels across countries

We compared the bisphenol levels observed in our study with those reported in four national surveys of other populations (routine monitoring programs) during a similar period of time. The comparison results are presented in Table 3. Taiwanese adults (20–39 years) at the 50th percentile of exposure (7.75 μg/L) exhibited more than five times the BPA levels of US (NHANES, 2013–2014) and Canada (CHMS, 2012–2013) adults of the same age (1.47 and 1.10 μg/L, respectively). In minors, the median levels of BPA observed in the present study and those observed in other countries can be ranked as follows: Taiwan (6–11 years: 4.83 μg/L; 12–19 years: 4.93 μg/L; the present study) > Canada [2012–2013; 6–11 years: 1.20 μg/L; 12–19 years: 1.40 μg/L; (69)] > United States [2013–2014; 6–11 years: 1.34 μg/L; 12–19 years: 1.14 μg/L; (32)].

## 4 Discussion

We discovered that the median concentration levels of BPs were higher in Taiwan than in other countries. Furthermore, the concentrations and DI of BPA and its substitutes in Taiwanese varied by age and sex. Notably, EFSA (46) re-established the TDI of BPA to 0.2 ng/kg/day. In our study, more than 99% of the 366 individuals had cumulate HI_BPs_ exceeding 1.

Compared to other studies (32, 70), we found that the median concentration level of BPA in Taiwanese adults was nearly 6-fold higher than in other countries (Taiwan [7.96 μg/L], USA [1.24 μg/L], and Korea [1.49 μg/L]), and creatinine-adjusted urinary BPA concentration was also higher than the finding of meta-analysis (age: >18 years old; totaling 28,353 participants) (present study (9.45) vs. meta-analysis (1.76) μg/g Cr) (71). In addition, in a study evaluating BPA concentrations in different Asian countries (adults of age ≥ 19, India [*n* = 21], Japan [*n* = 36]), Korea [*n* = 32], Kuwait [*n* = 32], Malaysia [*n* = 29], and Vietnam [*n* = 30]), the GM level (0.67 to 2.53 μg/g Cr) was approximately 4 to 9 times lower than our study (9.79 μg/g Cr) (72). Furthermore, the median levels of BPF and BPS for adults observed in our study (present study [7.89, 1.96] μg/L) were also 2- to 9-fold higher than those reported through the NHANES 2013–2014 (32) (USA [0.35, 0.37] μg/L), NHANES 2011–2016 (73) (USA [0.30, 0.50] μg/L) and the other study (Belgium [0.14, 0.11] μg/L). The above reasons may be caused by the exposure of dietary route (74) because the exposure of dietary BPA in Taiwan was approximately 100 to 400 times higher than in other countries (Taiwan: 133.79 to 419.26 ng/g (beef, chicken, and pork) vs. Norway: 0.24 ng/g (meat and meat products) (75, 76), indicating food insecurity as a predictor of high BPA exposure (77), and meat products are a major contributor (78). In addition, BPF and other BPA substitutes were also detected in foodstuffs (79). A US survey reported that BPF occurred as the second most abundant bisphenol substitute in a variety of food items (80). Bisphenol substitutes such as BPS and BPF are increasingly used to replace BPA (29), which can lead to their gradual accumulation in the environment. Hence, this may also explain why the concentrations of BPF and BPS are higher in Taiwan. In conclusion, the differences in demographic, dietary habits, and lifestyles may be responsible for the variation in population exposure levels of BPA and its substitute (81).

We found that bisphenol concentration levels and DI were higher in adults than in minors; it is consistent with the finding by Martín et al. (82) that BPA concentration levels were significantly higher in adults than in children, indicating that children are experiencing less exposure to BPA than adults (83). In addition, the median levels of bisphenols (creatinine-adjusted) in all participants were significantly increased along with increasing age; however, contradictory findings have been reported by other studies. Several previous studies point out that BPA and BPS exposure is typically higher in children than in adults (84, 85); the GM of BPA levels has been demonstrated to decrease with age (86), and the significant decrease in DI of BPA and BPS is observed with increasing age (14, 87). There are many reasons for elevated BPA and its substitute concentrations in adults. A possible explanation could be related to the more intense use of PCP products, especially the higher frequency of lotion use among adults. BPs are not used as ingredients in PCP formulations, but they are found in many products, such as shampoos, bath lotions, cosmetics, and other personal care products (41, 88, 89). Among the bisphenols analyzed, BPS was found across all the PCPs with the highest detection frequency of 71.5%, followed by BPF (56.7%) and BPA (57.7%) (88). Some studies point out that the urinary concentrations of BPA and BPS were not associated with the consumption patterns of cosmetics or PCPs (6, 90). However, in our study found that people who used lotion regularly had higher levels of bisphenols in their urine than those who used it less often, it consist with Yang finding’s, PCPs stored in packaging containing BPA could be subject to chemical leaching (91), facial moisturizer and body lotion were important contributors to BP exposure from PCPs both for men and women (29). Between the sexes, female individuals appear to be exposed to more BPs, which is consistent with other studies. BPA exposure has been reported to be common in women (4, 71). Otherwise, women use lotions more frequently, which may contribute to their higher exposure to BP levels. On the contrary, some studies (32, 87, 92) regarding the levels of bisphenol and its substitutes in different sex have reported inconsistent findings, indicating that the median concentrations and DI of BPA and its substitutes were statistically significantly higher in men than in women, and the DI of BPA and BPS was considerably greater in men than in women (14). These results suggest that the factors influencing exposure to BPA and its substitutes are intricate, with gender likely being just one of them (93).

The DIs of BPA and BPF by our participants were lower than the TDI of 4,000 ng/kg/day (65), whereas that of BPS were lower than the corresponding threshold of 4,400 ng/kg/day (64). Globally, BPA exposure was estimated at 60.08 ng/kg bw/day for children (94), while in Europe, the median of BPA DIs for children was approximately 46.3 ng/kg bw/day (95). In addition, the BPA intake levels in children in North America, Germany, and Asia were 46.64, 60, and 61.95 ng/kg bw/day, respectively (96, 97). The DI values of BPF and BPS (0.2 and 0.3 ng/kg/day, respectively) for school-age children (average age = 9.8 years) in Thailand (98) are similar to the findings of our study (0.77 and 0.24 ng/kg/day, respectively). According to NHANES (2005–2006, n = 2,638) findings, the DI of BPA in adults is approximately 34 ng/kg/day (87); similarly, another study found that the rank of DI in Europe is 18.1 to 39.5 ng/kg/day (95). Although the DI in our study (whether adults or minors) of BPA seems lower than above studies, the reason for this may be the differences in the method of daily intake calculated (including the difference of physiological index parameters) (99). Moreover, past studies rarely estimated the DI value of BPA substitutes such as BPF and BPS in the full general population. Hence, HQ and HI were calculated to quantitatively assess the potential health hazards of bisphenol which was necessary.

For each of the considered scenarios in our study, the calculated HQ value for individual compounds was found to be less than 1, which means that the intake of BPs was far below the RFD AA value (63) in a previous study and the TDI threshold was set by the EFSA (65); this finding is consistent with several other studies (81, 100–102). Nevertheless, in spite of no high risk associated with the HQ of BPA and its substitute, bisphenols exhibit that endocrine toxicity, neurotoxicity, and reproductive toxicity in preschoolers were non-negligible (100). According to that, the relevant HBGV must be updated to meet the current human health risk assessment standards (such as the TDI for the calculation of phenol-specific HQs should be updated) (101). Additionally, the HI values were < 1 (sum of the corresponding HQs of each bisphenol), and this finding is consistent with those of recent studies (33, 103), although some of these values were set many years ago (83).

Recently, EFSA (2023) re-evaluated the risks from BPA in foods and established a new TDI of 0.2 ng/kg/day, which was 20,000 times lower than the previous TDI of 4,000 ng/kg/day. Thus, we observed that nearly 95.6% of the 366 individuals in our study had an HI_BPA_ exceeding 1. When we assume that the TDI of BPF and BPS has the same TDI as BPA, more than 99% of the 366 individuals in our study had a cumulate HI_BPs_ value exceeding 1, indicating that bisphenol exposure risk was indeed a cause for concern; this finding was consistent with that of EFSA (2023), the dietary exposure to BPA is a major health concern for consumers of all ages. While most regulatory agencies and governments generally consider the current reference dose to be a “safe” threshold, more evidence shows adverse effects of BPA on human health at low exposure doses (83). We further found that the median values of HI were higher in adults than in minors; furthermore, the contribution of BPF to the total health hazard was higher than that of BPA in adults, indicating that we cannot ignore the BPA and its substitute in human effect.

However, the TDI and RfD for BPA substitutes remain unclear. Also, the safe levels of RfD or TDI for BPA and its substitutes were determined based on the general population and the anti-androgenic properties of animals, not on a specific age or gender. Therefore, the health hazards of the substitutes of BPA may be underestimated. In previous research (104), we suggested that even small doses of DEHP can cause infertility and ovarian toxicity in women of reproductive age. Consequently, governments across the world should not ignore the health concerns of BPA and its substitutes; relevant indicators for assessing health hazards (TDI or RfD) should be adjusted or set for different objects in the future, especially new substitutes of BPA. Notably, many countries have banned BPA in packaging, such as Spain (under a Law on Waste and Contaminated Soil) and France (under Law No. 2012–1,442). In contrast, Taiwan has been more reluctant in implementing BPA regulations, having only banned the use of BPA in baby bottles in 2013. According to TDI of EFSA (2023), the cumulative HI was higher in adults than in minors but higher in women than in men. Hence, it is suggested that the Taiwan government should face up to this problem to develop stricter regulations and educate the people in Taiwan to consciously choose products that are BPA-free (including BPF and BPS), avoid heating food in containers containing BPA, and refrain from storing warm food in such containers (105). At the same time, it is necessary to manage exposure sources based on different ages and genders. Expanding regulatory restrictions on the use of BPA-derived commodities is necessary, such as limiting the addition of BPA to personal care products and plastic utensils commonly used by women or restricting the use of BPA-containing materials in takeaway food packaging, especially restricting the use of BPA in baby/children products such as toys (106). Importantly, more research is needed to better understand the potential health effects of substitute exposure to BPA, especially in vulnerable populations such as pregnant women and children. This includes studies on the mechanisms of BPA substitute toxicity and the development of biomarkers for BPA substitute exposure.

The present study had several strengths. First, the data used in our study were collected from a representative survey of individuals aged 7–92 years; hence, our findings may represent the bisphenol exposure profiles of the Taiwanese population. Next, the level of daily creatinine excretion was estimated using several anthropometric indicators, which allowed us to assess the level of bisphenol exposure in a wide range of individuals, from minors to older adults. Finally, population-level exposure to BPA and its substitutes was estimated using urine samples; therefore, the risk assessment results may be more accurate than those obtained by accessing external exposure only. In addition, this study used samples collected when Taiwan banned the use of BPA in baby bottles, and the risk assessment results can be provided to the government as a reference.

Our study also had some limitations. First, the DIs of the three bisphenol compounds were calculated under the assumption that the spot urine samples were not of sufficiently high quality to represent the daily average urinary levels. Second, the study only assessed the frequency of use of PCPs and lacked information about the source of exposure to BPs from the diet. Based on past research (e.g., dietary sources), we can only explain why the general population of Taiwan has higher urinary BP concentration levels (creatinine-adjusted or unadjusted) than other countries. Third, in this study, the cumulative risk estimation was based only on the EFSA (2015, 2023) TDI and reference dose for anti-androgenicity, without assessment of health effects. In the future, we should use the various health-based guidance values to assess the different health effects as soon as possible.

## 5 Conclusion

In this study, 366 urinary levels of BPA and its substitutes were collected for general Taiwanese from various geographic regions. Our study revealed that the exposure profiles and risk of bisphenol and its substitutes in Taiwanese varied by age and sex. Notably, exposure levels of bisphenol and its substitutes were higher in Taiwan than in other counties. Our results also indicate that the exposure risk of BPA in Taiwan was deemed unacceptable according to the new EFSA regulations, and food contamination could be a possible source of exposure. We suggest that the risk of exposure to BPA and its substitutes in most human biomonitoring studies should be reassessed based on new scientific evidence. We also suggest that the government should ensure that products containing BPA are clearly labeled and encourage manufacturers to replace BPA with lower toxicity substances in their products in order to reduce BPA exposure for different age groups (e.g., toys and personal care products). Additionally, it is also recommended that the government take immediate action to implement stricter regulations on BPA substitutes.

## Data availability statement

The original contributions presented in the study are included in the article/Supplementary material, and further inquiries can be directed to the corresponding author.

## Ethics statement

The studies involving humans were approved by the study was approved by the Research Ethics Committee of National Health Research Institutes (No. EC1020206) of Taiwan. The studies were conducted in accordance with the local legislation and institutional requirements. Written informed consent for participation in this study was provided by the participants’ legal guardians/next of kin.

## Author contributions

Y-JL: Formal analysis, Writing – original draft. H-CC: Investigation, Methodology, Project administration, Supervision, Writing – review & editing. J-WC: Investigation, Project administration, Supervision, Writing – review & editing. H-BH: Investigation, Resources, Writing – review & editing. W-TC: Data curation, Investigation, Writing – review & editing. P-CH: Data curation, Funding acquisition, Investigation, Methodology, Project administration, Resources, Supervision, Writing – review & editing.

## References

[1] Park C Song H Choi J Sim S Kojima H Park J. The mixture effects of bisphenol derivatives on estrogen receptor and androgen receptor. Environ Pollut. (2020) 260:114036. doi: 10.1016/j.envpol.2020.114036, PMID: 31995776

[2] Hahladakis JN Iacovidou E Gerassimidou S. An overview of the occurrence, fate, and human risks of the bisphenol-a present in plastic materials, components, and products. Integr Environ Assess Manag. (2022) 19:45–62. doi: 10.1002/ieam.4611, PMID: 35362236

[3] Czarny-Krzymińska K Krawczyk B Szczukocki D. Bisphenol a and its substitutes in the aquatic environment: occurrence and toxicity assessment. Chemosphere. (2023) 315:137763. doi: 10.1016/j.chemosphere.2023.137763, PMID: 36623601

[4] Fisher M Arbuckle TE MacPherson S Braun JM Feeley M Gaudreau É. Phthalate and BPA exposure in women and newborns through personal care product use and food packaging. Environ Sci Technol. (2019) 53:10813–26. doi: 10.1021/acs.est.9b02372, PMID: 31424210

[5] Hwang M Choi K Park C. Urinary levels of phthalate, bisphenol, and paraben and allergic outcomes in children: Korean National Environmental Health survey 2015–2017. Sci Total Environ. (2022) 818:151703. doi: 10.1016/j.scitotenv.2021.151703, PMID: 34798094

[6] Lu S Yu Y Ren L Zhang X Liu G Yu Y. Estimation of intake and uptake of bisphenols and triclosan from personal care products by dermal contact. Sci Total Environ. (2018) 621:1389–96. doi: 10.1016/j.scitotenv.2017.10.088, PMID: 29054660

[7] Tarafdar A Sirohi R Balakumaran PA Reshmy R Madhavan A Sindhu R. The hazardous threat of bisphenol a: toxicity, detection and remediation. J Hazard Mater. (2022) 423:127097. doi: 10.1016/j.jhazmat.2021.127097, PMID: 34488101

[8] Ginter-Kramarczyk D Zembrzuska J Kruszelnicka I Zając-Woźnialis A Ciślak M. Influence of temperature on the quantity of bisphenol a in bottled drinking water. Int J Environ Health Res. (2022) 19:5710. doi: 10.3390/ijerph19095710 PMC9104415 35565103

[9] Ugboka UG Ihedioha JN Ekere NR Okechukwu FO. Human health risk assessment of bisphenol a released from polycarbonate drinking water bottles and carbonated drinks exposed to sunlight in Nigeria. Int J Environ Anal Chem. (2020) 102:2830–40. doi: 10.1080/03067319.2020.1759572

[10] Hartle JC Zawadzki RS Rigdon J Lam J Gardner CD. Development and evaluation of a novel dietary bisphenol a (BPA) exposure risk tool. BMC Nutr. (2022) 8:143. doi: 10.1186/s40795-022-00634-4, PMID: 36474269 PMC9724381

[11] Herrero M Souza MC González N Marquès M Barbosa F Domingo JL. Dermal exposure to bisphenols in pregnant women's and baby clothes: risk characterization. Sci Total Environ. (2023) 878:163122. doi: 10.1016/j.scitotenv.2023.163122, PMID: 37001656

[12] Márton É Varga A Penyige A Birkó Z Balogh I Nagy B. Comparative analysis of transcriptomic changes including mrna and microrna expression induced by the xenoestrogens Zearalenone and bisphenol a in human ovarian cells. Toxins (Basel). (2023) 15:140. doi: 10.3390/toxins15020140, PMID: 36828454 PMC9967916

[13] Russo G Barbato F Mita DG Grumetto L. Occurrence of bisphenol a and its analogues in some foodstuff marketed in Europe. Food Chem Toxicol. (2019) 131:110575. doi: 10.1016/j.fct.2019.110575, PMID: 31201899

[14] Wang H Gao R Liang W Wei S Zhou Y Zeng F. Assessment of BPA and BPS exposure in the general population in Guangzhou, China-estimation of daily intakes based on urinary metabolites. Environ Pollut. (2022) 315:120375. doi: 10.1016/j.envpol.2022.120375, PMID: 36220574

[15] Huang X Cang X Liu J. Molecular mechanism of bisphenol a on androgen receptor antagonism. Toxicol In Vitro. (2019) 61:104621. doi: 10.1016/j.tiv.2019.104621, PMID: 31415812

[16] Buoso E Masi M Racchi M Corsini E. Endocrine-disrupting Chemicals' (EDCs) effects on tumour microenvironment and Cancer progression: emerging contribution of RACK1. Int J Mol Sci. (2020) 21:9229. doi: 10.3390/ijms21239229, PMID: 33287384 PMC7729595

[17] Molina-López AM Bujalance-Reyes F Ayala-Soldado N Mora-Medina R Lora-Benítez A Moyano-Salvago R. An overview of the health effects of bisphenol a from a one health perspective. Animals (Basel). (2023) 13:2439. doi: 10.3390/ani13152439, PMID: 37570248 PMC10417040

[18] Huang YQ Wong CKC Zheng JS Bouwman H Barra R Wahlström B. Bisphenol a (BPA) in China: a review of sources, environmental levels, and potential human health impacts. Environ Int. (2012) 42:91–9. doi: 10.1016/j.envint.2011.04.010, PMID: 21596439

[19] Ma Y Liu H Wu J Yuan L Wang Y Du X. The adverse health effects of bisphenol a and related toxicity mechanisms. Environ Res. (2019) 176:108575. doi: 10.1016/j.envres.2019.108575 31299621

[20] Buoso E Kenda M Masi M Linciano P Galbiati V Racchi M. Effects of bisphenols on RACK1 expression and their immunological implications in THP-1 cells. Front Pharmacol. (2021) 12:743991. doi: 10.3389/fphar.2021.743991, PMID: 34621174 PMC8490885

[21] Cariati F Carbone L Conforti A Bagnulo F Peluso SR Carotenuto C. Bisphenol A-induced epigenetic changes and its effects on the male reproductive system. Front Endocrinol (Lausanne). (2020) 11:453. doi: 10.3389/fendo.2020.00453, PMID: 32849263 PMC7406566

[22] Kataria N Bhushan D Gupta R Rajendran S Teo MY Khoo KS. Current progress in treatment technologies for plastic waste (bisphenol a) in aquatic environment: occurrence, toxicity and remediation mechanisms. Environ Pollut. (2022) 315:120319. doi: 10.1016/j.envpol.2022.120319, PMID: 36183872

[23] Matuszczak E Komarowska MD Debek W Hermanowicz A. The impact of bisphenol a on fertility, reproductive system, and development: a review of the literature. Int J Endocrinol. (2019) 2019:4068717. doi: 10.1155/2019/4068717, PMID: 31093279 PMC6481157

[24] Nowak K Jabłońska E Ratajczak-Wrona W. Immunomodulatory effects of synthetic endocrine disrupting chemicals on the development and functions of human immune cells. Environ Int. (2019) 125:350–64. doi: 10.1016/j.envint.2019.01.078, PMID: 30743143

[25] Canada-Gazette. (2010). Part II 144 (21). Available at: https://canadagazette.gc.ca/rp-pr/p2/2010/2010-10-13/pdf/g2-14421.pdf (Accessed October 13, 2010)

[26] Flint S Markle T Thompson S Wallace E. Bisphenol a exposure, effects, and policy: a wildlife perspective. J Environ Manag. (2012) 104:19–34. doi: 10.1016/j.jenvman.2012.03.021, PMID: 22481365

[27] Bornehag CG Engdahl E Unenge Hallerbäck M Wikström S Lindh C Rüegg J. Prenatal exposure to bisphenols and cognitive function in children at 7 years of age in the Swedish SELMA study. Environ Int. (2021) 150:106433. doi: 10.1016/j.envint.2021.106433, PMID: 33637302

[28] Pang Q Li Y Meng L Li G Luo Z Fan R. Neurotoxicity of BPA, BPS, and BPB for the hippocampal cell line (HT-22): an implication for the replacement of BPA in plastics. Chemosphere. (2019) 226:545–52. doi: 10.1016/j.chemosphere.2019.03.177, PMID: 30953899

[29] Karrer C Andreassen M von Goetz N Sonnet F Sakhi AK Hungerbühler K. The EuroMix human biomonitoring study: source-to-dose modeling of cumulative and aggregate exposure for the bisphenols BPA, BPS, and BPF and comparison with measured urinary levels. Environ Int. (2020) 136:105397. doi: 10.1016/j.envint.2019.105397, PMID: 31884417

[30] Gys C Ait Bamai Y Araki A Bastiaensen M Caballero-Casero N Kishi R. Biomonitoring and temporal trends of bisphenols exposure in Japanese school children. Environ Res. (2020) 191:110172. doi: 10.1016/j.envres.2020.110172, PMID: 32919958

[31] Health Canada (2021). Bisphenol A (BPA) in Canadians. Available at: https://www.canada.ca/content/dam/hc-sc/documents/services/environmental-workplace-health/reports-publications/environmental-contaminants/human-biomonitoring-resources/bisphenol-a-canadians/bpa-eng.pdf (Accessed December 14, 2021)

[32] Lehmler HJ Liu B Gadogbe M Bao W. Exposure to bisphenol A, bisphenol F, and Bisphenol S in U.S. adults and children: the National Health and Nutrition Examination Survey. ACS Omega. (2018, 2013–2014) 3:6523–32. doi: 10.1021/acsomega.8b00824, PMID: 29978145 PMC6028148

[33] Lyu Z Harada KH Kim S Fujitani T Hitomi T Pan R. Temporal trends in bisphenol exposures and associated health risk among Japanese women living in the Kyoto area from 1993 to 2016. Chemosphere. (2023) 316:137867. doi: 10.1016/j.chemosphere.2023.137867, PMID: 36642136

[34] Tang S He C Thai PK Heffernan A Vijayasarathy S Toms L. Urinary concentrations of bisphenols in the Australian population and their association with the per capita mass loads in wastewater. Environ Sci Technol. (2020) 54:10141–8. doi: 10.1021/acs.est.0c00921, PMID: 32806918

[35] Qiu W Liu S Chen H Luo S Xiong Y Wang X. The comparative toxicities of BPA, BPB, BPS, BPF, and BPAF on the reproductive neuroendocrine system of zebrafish embryos and its mechanisms. J Hazard Mater. (2021) 406:124303. doi: 10.1016/j.jhazmat.2020.124303, PMID: 33121856

[36] Gély CA Lacroix MZ Roques BB Toutain P-L Gayrard V Picard-Hagen N. Comparison of toxicokinetic properties of eleven analogues of bisphenol a in pig after intravenous and oral administrations. Environ Int. (2023) 171:107722. doi: 10.1016/j.envint.2022.107722, PMID: 36584424

[37] Karrer C de Boer W Delmaar C Cai Y Crépet A Hungerbühler K. Linking probabilistic exposure and pharmacokinetic modeling to assess the cumulative risk from the bisphenols BPA, BPS, BPF, and BPAF for Europeans. Environ Sci Technol. (2019) 53:9181–91. doi: 10.1021/acs.est.9b01749, PMID: 31294980

[38] Nevoral J Kolinko Y Moravec J Žalmanová T Hošková K Prokešová Š. Long-term exposure to very low doses of bisphenol S affects female reproduction. Chemosphere. (2018) 156:47–57. doi: 10.1530/REP-18-0092, PMID: 29748175

[39] Thoene M Dzika E Gonkowski S Wojtkiewicz J. Bisphenol S in food causes hormonal and obesogenic effects comparable to or worse than bisphenol a: A literature review. Nutrients. (2020) 12:532. doi: 10.3390/nu12020532, PMID: 32092919 PMC7071457

[40] Kiook B Jong-Tae P Kyeongmin K. Association of Urinary Bisphenols Concentration with asthma in Korean adolescents: data from the third Korean National Environmental Health Survey. Toxic. (2021) 9:291. doi: 10.3390/toxics9110291, PMID: 34822682 PMC8621547

[41] Martín-Pozo L Gómez-Regalado M Moscoso-Ruiz I Zafra-Gómez A. Analytical methods for the determination of endocrine disrupting chemicals in cosmetics and personal care products: a review. Talanta. (2021) 234:122642. doi: 10.1016/j.talanta.2021.122642, PMID: 34364451

[42] Moreno-Gómez-Toledano R. Relationship between emergent BPA-substitutes and renal and cardiovascular diseases in adult population. Environ Pollut. (2022) 313:120106. doi: 10.1016/j.envpol.2022.120106, PMID: 36084738

[43] Martínez MÁ Blanco J Rovira J Kumar V Domingo JL Schuhmacher M. Bisphenol a analogues (BPS and BPF) present a greater obesogenic capacity in 3T3-L1 cell line. Food Chem Toxicol. (2020) 140:111298. doi: 10.1016/j.fct.2020.111298, PMID: 32220626

[44] Beronius A Rudén C Håkansson H Hanberg A. Risk to all or none? A comparative analysis of controversies in the health risk assessment of bisphenol a. Reprod Toxicol. (2010) 29:132–46. doi: 10.1016/j.reprotox.2009.11.007 19931376

[45] Hwang M Park SJ Lee HJ. Risk assessment of bisphenol a in the Korean general population. Appl Sci. (2023) 13:3587. doi: 10.3390/app13063587

[46] European Food Safety Authority. Re-evaluation of the risks to public health related to the presence of bisphenol a (BPA) in foodstuffs. EFSA J. (2023) 21:6857. doi: 10.2903/j.efsa.2023.6857, PMID: 37089179 PMC10113887

[47] Eladak S Grisin T Moison D Guerquin MJ N'Tumba-Byn T Pozzi-Gaudin S. A new chapter in the bisphenol a story: bisphenol S and bisphenol F are not safe alternatives to this compound. Fertil Steril. (2015) 103:11–21. doi: 10.1016/j.fertnstert.2014.11.005, PMID: 25475787

[48] Chang JW Liao KW Huang CY Huang HB Chang WT Jaakkola JJK. Phthalate exposure increased the risk of early renal impairment in Taiwanese without type 2 diabetes mellitus. Int J Hyg Environ Health. (2020) 224:113414. doi: 10.1016/j.ijheh.2019.10.009, PMID: 31784327

[49] Huang HB Siao CY Lo YC Shih SF Lu CH Huang PC. Mediation effects of thyroid function in the associations between phthalate exposure and glucose metabolism in adults. Environ Pollut. (2021) 278:116799. doi: 10.1016/j.envpol.2021.116799, PMID: 33743268

[50] Huang PC Chen HC Chou WC Lin HW Chang WT Chang JW. Cumulative risk assessment and exposure characteristics of parabens in the general Taiwanese using multiple hazard indices approaches. Sci Total Environ. (2022) 843:156821. doi: 10.1016/j.scitotenv.2022.156821, PMID: 35738379

[51] Huang PC Tsai CH Liang WY Li SS Pan WH Chiang HC. Age and gender differences in urinary levels of eleven phthalate metabolites in general Taiwanese population after a dehp episode. PLoS One. (2015) 10:e0133782. doi: 10.1371/journal.pone.0133782, PMID: 26207744 PMC4514596

[52] Huang PC Waits A Chen HC Chang WT Jaakkola JJK Huang HB. Mediating role of oxidative/nitrosative stress biomarkers in the associations between phthalate exposure and thyroid function in Taiwanese adults. Environ Int. (2020) 140:105751. doi: 10.1016/j.envint.2020.105751, PMID: 32353668

[53] Chen HC Chang JW Sun YC Chang WT Huang PC. Determination of parabens, bisphenol a and its analogs, Triclosan, and Benzophenone-3 levels in human urine by isotope-dilution-UPLC-MS/MS method followed by supported liquid extraction. Toxics. (2022) 10:21. doi: 10.3390/toxics10010021, PMID: 35051063 PMC8781104

[54] EMA. Guideline on bioanalytical method validation. London, United Kingdom: European Union (2011).

[55] Chang JW Lee CC Pan WH Chou WC Huang HB Chiang HC. Estimated daily intake and cumulative risk assessment of phthalates in the general Taiwanese after the 2011 DEHP food scandal. Sci Rep. (2017) 7:45009. doi: 10.1038/srep45009, PMID: 28327585 PMC5361203

[56] Kortenkamp A Scholze M Ermler S Priskorn L Jørgensen N Andersson AM. Combined exposures to bisphenols, polychlorinated dioxins, paracetamol, and phthalates as drivers of deteriorating semen quality. Environ Int. (2022) 165:107322. doi: 10.1016/j.envint.2022.107322, PMID: 35691715

[57] Liu J Wattar N Field CJ Dinu I Dewey D Martin JW. Exposure and dietary sources of bisphenol a (BPA) and BPA-alternatives among mothers in the apron cohort study. Environ Int. (2018) 119:319–26. doi: 10.1016/j.envint.2018.07.001, PMID: 29990952

[58] Thayer KA Doerge DR Hunt D Schurman SH Twaddle NC Churchwell MI. Pharmacokinetics of bisphenol a in humans following a single oral administration. Environ Int. (2015) 83:107–15. doi: 10.1016/j.envint.2015.06.008, PMID: 26115537 PMC4545316

[59] Völkel W Colnot T Csanády GA Filser JG Dekant W. Metabolism and kinetics of bisphenol a in humans at low doses following oral administration. Chem Res Toxicol. (2002) 15:1281–7. doi: 10.1021/tx025548t, PMID: 12387626

[60] Mage DT Allen RH Gondy G Smith W Barr DB Needham LL. Estimating pesticide dose from urinary pesticide concentration data by creatinine correction in the third national health and nutrition examination survey (NHANES-III). J Expo Sci Environ Epidemiol. (2004) 14:457–65. doi: 10.1038/sj.jea.7500343, PMID: 15367927

[61] Mage DT Allen RH Kodali A. Creatinine corrections for estimating children’s and adult’s pesticide intake doses in equilibrium with urinary pesticide and creatinine concentrations. J Expo Sci Environ Epidemiol. (2008) 18:360–8. doi: 10.1038/sj.jes.7500614, PMID: 17878925

[62] Cunha SC Inácio T Almada M Ferreira R Fernandes JO. Gas chromatography-mass spectrometry analysis of nine bisphenols in canned meat products and human risk estimation. Food Res Int. (2020) 135:109293. doi: 10.1016/j.foodres.2020.109293, PMID: 32527484

[63] Kortenkamp A Faust M. Combined exposures to anti-androgenic chemicals: steps towards cumulative risk assessment. Int J Androl. (2010) 33:463–74. doi: 10.1111/j.1365-2605.2009.01047.x, PMID: 20487045

[64] Mok S Jeong Y Park M Kim S Lee I Park J. Exposure to phthalates and bisphenol analogues among childbearing-aged women in Korea: influencing factors and potential health risks. Chemosphere. (2021) 264:128425. doi: 10.1016/j.chemosphere.2020.128425 33010629

[65] European Food Safety Authority. Scientific opinion on the risks to public health related to the presence of bisphenol a (BPA) in foodstuffs. EFSA J. (2015) 13:3978. doi: 10.2903/j.efsa.2015.3978 PMC10113887 37089179

[66] Lin N Ma D Liu Z Wang X Ma L. Migration of bisphenol a and its related compounds in canned seafood and dietary exposure estimation. Food Quality and Safety. (2022) 6:fyac006. doi: 10.1093/fqsafe/fyac006

[67] Strømmen K Lyche JL Moltu SJ Müller MHB Blakstad EW Brække K. Estimated daily intake of phthalates, parabens, and bisphenol a in hospitalised very low birth weight infants. Chemosphere. (2022) 309:136687. doi: 10.1016/j.chemosphere.2022.136687, PMID: 36206919

[68] Derakhshan A Philips EM Ghassabian A Santos S Asimakopoulos AG Kannan K. Association of urinary bisphenols during pregnancy with maternal, cord blood and childhood thyroid function. Environ Int. (2021) 146:106160. doi: 10.1016/j.envint.2020.106160, PMID: 33068853

[69] Health Canada. (2015). Bisphenol A concentrations in Canadians, 2012 and 2013. Available at: https://www150.statcan.gc.ca/n1/pub/82-625-x/2015001/article/14208-eng.htm (Accessed November 27, 2015)

[70] Park C Hwang M Baek Y Jung S Lee Y Paek D. Urinary phthalate metabolite and bisphenol a levels in the Korean adult population in association with sociodemographic and behavioral characteristics: Korean National Environmental Health Survey (KONEHS) 2012–2014. Int J Hyg Environ Health. (2019) 222:903–10. doi: 10.1016/j.ijheh.2019.02.003, PMID: 30773337

[71] Colorado-Yohar SM Castillo-González AC Sánchez-Meca J Rubio-Aparicio M Sánchez-Rodríguez D Salamanca-Fernández E. Concentrations of bisphenol-a in adults from the general population: a systematic review and meta-analysis. Sci Total Environ. (2021) 775:145755. doi: 10.1016/j.scitotenv.2021.145755, PMID: 34132197

[72] Zhang Z Alomirah H Cho H-S Li Y-F Liao C Minh TB. Urinary bisphenol a concentrations and their implications for human exposure in several Asian countries. Environ Sci Technol. (2011) 45:7044–50. doi: 10.1021/es200976k, PMID: 21732633

[73] Zhang C Luo Y Qiu S Huang X Jin K Li J. Associations between urinary concentrations of bisphenols and serum concentrations of sex hormones among US. Males Environ Health. (2022) 21:135. doi: 10.1186/s12940-022-00949-6, PMID: 36550468 PMC9773582

[74] Geens T Aerts D Berthot C Bourguignon J-P Goeyens L Lecomte P. A review of dietary and non-dietary exposure to bisphenol-a. Food Chem Toxicol. (2012) 50:3725–40. doi: 10.1016/j.fct.2012.07.059, PMID: 22889897

[75] Chen WY Shen YP Chen SC. Assessing bisphenol a (BPA) exposure risk from long-term dietary intakes in Taiwan. Sci Total Environ. (2016) 543:140–6. doi: 10.1016/j.scitotenv.2015.11.029, PMID: 26580736

[76] Sakhi AK Lillegaard IT Voorspoels S Carlsen MH Løken EB Brantsæter AL. Concentrations of phthalates and bisphenol a in Norwegian foods and beverages and estimated dietary exposure in adults. Environ Int. (2014) 73:259–69. doi: 10.1016/j.envint.2014.08.005, PMID: 25173060

[77] van Woerden I Bruening M Montresor-López J Payne-Sturges DC. Trends and disparities in urinary BPA concentrations among U.S. emerging adults. Environ Res. (2019) 176:108515. doi: 10.1016/j.envres.2019.05.046, PMID: 31195292

[78] Wang X Nag R Brunton NP Siddique MA Harrison SM Monahan FJ. Human health risk assessment of bisphenol a (BPA) through meat products. Environ Res. (2022) 213:113734. doi: 10.1016/j.envres.2022.113734, PMID: 35750124

[79] Cao XL Kosarac I Popovic S Zhou S Smith D Dabeka R. LC-MS/MS analysis of bisphenol S and five other bisphenols in total diet food samples. Food Addit Contam Part A Chem Anal Control Expo Risk Assess. (2019) 36:1740–7. doi: 10.1080/19440049.2019.1643042, PMID: 31361189

[80] Liao C Kannan K. Concentrations and profiles of bisphenol a and other bisphenol analogues in foodstuffs from the United States and their implications for human exposure. J Agric Food Chem. (2013) 61:4655–62. doi: 10.1021/jf400445n, PMID: 23614805

[81] Cui FP Yang P Liu C Chen PP Deng YL Miao Y. Urinary bisphenol a and its alternatives among pregnant women: predictors and risk assessment. Sci Total Environ. (2021) 784:147184. doi: 10.1016/j.scitotenv.2021.147184 33901963

[82] Martín J Santos JL Aparicio I Alonso E. Exposure assessment to parabens, bisphenol a and perfluoroalkyl compounds in children, women and men by hair analysis. Sci Total Environ. (2019) 695:133864. doi: 10.1016/j.scitotenv.2019.133864, PMID: 31421338

[83] Dueñas-Moreno J Mora A Kumar M Meng XZ Mahlknecht J. Worldwide risk assessment of phthalates and bisphenol a in humans: the need for updating guidelines. Environ Int. (2023) 181:108294. doi: 10.1016/j.envint.2023.108294, PMID: 37935082

[84] Chen Y Fang J Ren L Fan R Zhang J Liu G. Urinary bisphenol analogues and triclosan in children from South China and implications for human exposure. Environ Pollut. (2018) 238:299–305. doi: 10.1016/j.envpol.2018.03.031, PMID: 29573712

[85] Heffernan AL Aylward LL Toms LM Eaglesham G Hobson P Sly PD. Age-related trends in urinary excretion of bisphenol a in Australian children and adults: evidence from a pooled sample study using samples of convenience. J Toxicol Environ Health A. (2013) 76:1039–55. doi: 10.1080/15287394.2013.834856, PMID: 24188190

[86] Jung SK Choi W Kim SY Hong S Jeon HL Joo Y. Profile of environmental chemicals in the Korean population—results of the Korean National Environmental Health Survey (KONEHS) cycle 3, 2015–2017. Int J Environ Res Public Health. (2022) 19:626. doi: 10.3390/ijerph19020626, PMID: 35055445 PMC8776061

[87] LaKind JS Naiman DQ. Daily intake of bisphenol a and potential sources of exposure: 2005–2006 National Health and nutrition examination survey. J Expo Sci Environ Epidemiol. (2010) 21:272–9. doi: 10.1038/jes.2010.9, PMID: 20237498 PMC3079892

[88] Jala A Varghese B Dutta R Adela R Borkar RM. Levels of parabens and bisphenols in personal care products and urinary concentrations in Indian young adult women: implications for human exposure and health risk assessment. Chemosphere. (2022) 297:134028. doi: 10.1016/j.chemosphere.2022.134028, PMID: 35218786

[89] Terry P. Chen J. (2012) Faculty opinions recommendation of endocrine disruptors and asthma-associated chemicals in consumer products. Faculty Opinions–Post-Publication Peer Review of the Biomedical Literature 10.1289/ehp.1104052 PMC3404651 22398195

[90] Liao C Kannan K. A survey of alkylphenols, bisphenols, and triclosan in personal care products from China and the United States. Arch Environ Contam Toxicol. (2014) 67:50–9. doi: 10.1007/s00244-014-0016-8, PMID: 24639116

[91] Yang CZ Yaniger SI Jordan VC Klein DJ Bittner GD. Most plastic products release estrogenic chemicals: a potential health problem that can be solved. Environ Health Perspect. (2011) 119:989–96. doi: 10.1289/ehp.1003220, PMID: 21367689 PMC3222987

[92] LaKind JS Naiman DQ. Temporal trends in bisphenol a exposure in the United States from 2003–2012 and factors associated with BPA exposure: spot samples and urine dilution complicate data interpretation. Environ Res. (2015) 142:84–95. doi: 10.1016/j.envres.2015.06.013, PMID: 26121292

[93] Robles-Aguilera V Gálvez-Ontiveros Y Rodrigo L Salcedo-Bellido I Aguilera M Zafra-Gómez A. Factors associated with exposure to dietary bisphenols in adolescents. Nutrients. (2021) 13:1553. doi: 10.3390/nu13051553, PMID: 34062990 PMC8147950

[94] Huang RP Liu ZH Yuan SF Yin H Dang Z Wu PX. Worldwide human daily intakes of bisphenol a (BPA) estimated from global urinary concentration data (2000-2016) and its risk analysis. Environ Pollut. (2017) 230:143–52. doi: 10.1016/j.envpol.2017.06.026, PMID: 28649042

[95] Garí M Moos R Bury D Kasper-Sonnenberg M Jankowska A Andysz A. Human-biomonitoring derived exposure and daily intakes of bisphenol a and their associations with neurodevelopmental outcomes among children of the polish mother and child cohort study. Erratum in: Environ Health. (2021) 20:95. doi: 10.1186/s12940-021-00777-0 PMC8390261 34433458

[96] Becker K Göen T Seiwert M Conrad A Pick-Fus H Müller J. GerES IV: phthalate metabolites and bisphenol a in urine of German children. Int J Hyg Environ Health. (2009) 212:685–92. doi: 10.1016/j.ijheh.2009.08.002, PMID: 19729343

[97] Huang RP Liu ZH Yin H Dang Z Wu PX Zhu NW. Bisphenol a concentrations in human urine, human intakes across six continents, and annual trends of average intakes in adult and child populations worldwide: a thorough literature review. Sci Total Environ. (2018) 626:971–81. doi: 10.1016/j.scitotenv.2018.01.144, PMID: 29898562

[98] Numsriskulrat N Teeranathada T Bongsebandhu-Phubhakdi C Aroonparkmongkol S Choi K Supornsilchai V. Exposure to bisphenol a and its analogs among Thai school-age children. Toxics. (2023) 11:761. doi: 10.3390/toxics11090761, PMID: 37755771 PMC10536550

[99] Çiftçi S Yalçın SS Samur G. Comparison of daily bisphenol a intake based on dietary and urinary levels in breastfeeding women. Reprod Toxicol. (2021) 106:9–17. doi: 10.1016/j.reprotox.2021.09.011, PMID: 34563571

[100] Fan D Liang M Guo M Gu W Gu J Liu M. Exposure of preschool-aged children to highly-concerned bisphenol analogues in Nanjing. East China Ecotoxicol Environ Saf. (2022) 234:113397. doi: 10.1016/j.ecoenv.2022.113397, PMID: 35286960

[101] Li R Zhan W Ren J Zhang F Huang X Ma Y. Temporal trends in risk of bisphenol a, benzophenone-3 and triclosan exposure among U.S. children and adolescents aged 6–19 years: findings from the National Health and nutrition examination survey 2005–2016. Environ Res. (2023) 216:114474. doi: 10.1016/j.envres.2022.114474, PMID: 36202243

[102] Liu Y Yan Z Zhang Q Song N Cheng J Torres OL. Urinary levels, composition profile and cumulative risk of bisphenols in preschool-aged children from Nanjing suburb. China Ecotoxicol Environ Saf. (2019) 172:444–50. doi: 10.1016/j.ecoenv.2019.02.002, PMID: 30735977

[103] Chang WH Liu SC Chen HL Lee CC. Dietary intake of 4-nonylphenol and bisphenol a in Taiwanese population: integrated risk assessment based on probabilistic and sensitive approach. Environ Pollut. (2019) 244:143–52. doi: 10.1016/j.envpol.2018.10.040, PMID: 30326386

[104] Chang WH Chou WC Waits A Liao KW Kuo PL Huang PC. Cumulative risk assessment of phthalates exposure for recurrent pregnancy loss in reproductive-aged women population using multiple hazard indices approaches. Environ Int. (2021) 154:106657. doi: 10.1016/j.envint.2021.106657, PMID: 34052604

[105] Sokal A Jarmakiewicz-Czaja S Tabarkiewicz J Filip R. Dietary intake of endocrine disrupting substances presents in environment and their impact on thyroid function. Nutrients. (2021) 13:867. doi: 10.3390/nu13030867, PMID: 33800806 PMC7998837

[106] Tsai WT. Survey on the environmental risks of bisphenol a and its relevant regulations in Taiwan: an environmental endocrine-disrupting chemical of increasing concern. Toxics. (2023) 11:722. doi: 10.3390/toxics11090722, PMID: 37755733 PMC10535487

[107] Tschersich C Murawski A Schwedler G Rucic E Moos RK Kasper-Sonnenberg M. Bisphenol a and six other environmental phenols in urine of children and adolescents in Germany – human biomonitoring results of the German environmental survey 2014–2017 (geres V). Sci Total Environ. (2021) 763:144615. doi: 10.1016/j.scitotenv.2020.144615, PMID: 33383503

