# Supplementary material for: Exposure characteristics and cumulative risk assessment of bisphenol A and its substitutes: the Taiwan environmental survey for toxicants 2013
Source: Front Public Health. 2024 May 23;12:1396147. doi: 10.3389/fpubh.2024.1396147 (PMC11153798; doi:10.3389/fpubh.2024.1396147)
Supplement: Supplementary file 1 [file Data_Sheet_1.docx]

**Supplementary material**

Exposure Characteristics and Cumulative Risk Assessment of Bisphenol A and Its Substitutes: The Taiwan Environmental Survey for Toxicants (TEST) 2013

Yu-Jung Lin ^a^, Hsin-Chang Chen ^b^, Jung-Wei Chang ^c^, Han-Bin Huang ^d^, Wan-Ting Chang ^a^, Po-Chin Huang ^a, e, f, g, ⁎^

^a^ National Institute of Environmental Health Sciences, National Health Research Institutes, Miaoli, Taiwan

^b^ Department of Chemistry, Tunghai University, Taichung, Taiwan

^c^ Institute of Environmental and Occupational Health Sciences, School of Medicine, National Yang Ming Chiao Tung University, Taipei, Taiwan

^d^ School of Public Health, National Defense Medical Center, Taipei, Taiwan

^e^ Research Center for Precision Environmental Medicine, Kaohsiung Medical University, Kaohsiung, Taiwan

^f^ Department of Medical Research, China Medical University Hospital, China Medical University, Taichung, Taiwan

^g^ Department of Safety, Health and Environmental Engineering, National United University, Miaoli, Taiwan

**Corresponding Author (*)**:

Po-Chin Huang, PhD, Associate Investigator,

National Institute of Environmental Health Sciences,

National Health Research Institutes, 35 Keyan Road, Zhunan, Miaoli County, 35035,

Email: [pchuang@nhri.edu.tw](mailto:pchuang@nhri.edu.tw)

Supplementary Information- QA/QC for Materials and Methods

The proposed method was validated according to guidelines established in the EMA (European Medicines Agency).Based on the guidance of EMA, the concentration points of the three QC levels, which should not be the concentration points of the calibrators, were 3 × LLOQ (lower limits of quantification), 30–50% of ULOQ (upper limit of quantification) and ≥75% of ULOQ . At intervals of every 10 samples, three spiked QC samples with three concentrations were applied during sample analysis to determine within-run and between-run assay variability. According to the Quality Control Criteria of EMA, the accuracy criteria was ≥ 85%, and the precision criteria were ≤ 15% (EMA 2011).

The SLE (supported liquid extraction) technique can provides acceptable recovery of target analytes with minimal interference in LC-MS (Liquid Chromatography Mass Spectrometry) bioanalysis (Hurtado-Sánchez et al. 2015; Liu et al. 2015; Zhongzhe and Jiang 2019). The matrix effect was examined to discuss the removal of interference. Table S1 lists the observed results of recovery, and the matrix effect of bisphenol in artificial urine, which shows acceptable and stable recoveries. The mean recoveries (%RSD) of bisphenol in low-, median-, and high-level concentrations were 75.6%–85.0% (1.8%–3.8%), 89.1%–93.0% (4.3%–8.6%), and 90.0%–93.7% (8.4%–6.2%), respectively. The recovery of three bisphenols achieved the criteria of EMA. The mean matrix effects for three levels of analytes spiked in artificial urine ranged from 79.5% to 103.2%, and the precisions (%RSD), measured at < 14.2%, met the criteria of EMA (15% for the precision of matrix effect) (EMA 2011).

Regarding the within-run and between-run accuracy and precision, the within-run accuracies (precision) for low-, median-, and high-level concentrations were 88.1%–110.4% (precision ≤ 11.7%), 97.4%–112.4% (precision ≤ 8.8%), and 99.8%–110.1% (precision ≤ 5.6%), respectively; the between-run accuracies (precision) for low-, median-, and high-level concentration were 98.2%–104.4% (precision ≤ 6.3%), 100.5%–105.8% (precision ≤ 4.2%), and 100.4–101.0% (precision ≤ 3.0%), respectively (Table S2). The results of the within-run and between-run accuracy and precision satisfied the criteria of EMA to determine the target bisphenols in human urine.

Table S3 lists the LOD (Limits of detection) and LLOQ (lower limits of quantification) of each bisphenol evaluated with the spiked EDCs in artificial urine followed by the SLE-technique extraction were 0.1 and 0.3 ng/mL, respectively, and the ULOQ (upper limit of quantification) of bisphenols were selected according to their distributions in human urine. The ULOQs for BPA and its substitutes (BPF, BPS) were 500 ng/mL.

**Table S1** Recovery and matrix effect of target parabens spike in artificial urine.

| Analyte | Spike Conc.  (ng/mL) | Recovery (n = 3) | | Matrix Effect (n = 3) | |
| --- | --- | --- | --- | --- | --- |
|  |  | Mean | RSD | Mean | RSD |
| BPA | 0.9 | 85.0% | 3.8% | 95.3% | 5.1% |
|  | 250 | 89.1% | 4.3% | 79.5% | 5.5% |
|  | 375 | 93.7% | 6.2% | 81.4% | 5.0% |
| BPF | 0.9 | 75.6% | 1.8% | 103.2% | 4.0% |
|  | 250 | 93.0% | 8.6% | 81.3% | 3.7% |
|  | 375 | 90.0% | 8.4% | 86.3% | 4.7% |
| BPS | 0.9 | 81.0% | 4.6% | 99.0% | 6.4% |
|  | 250 | 89.4% | 11.1% | 87.7% | 0.6% |
|  | 375 | 90.5% | 5.6% | 87.3% | 0.3% |

**Table S2** Intraday and interday accuracy and precision.

| **Analyte** | **Spiked Conc.**  **(ng/mL)** | **Within-Run (*n* = 5)** | | | **Between-Run (*n* = 5 × 3)** | | |
| --- | --- | --- | --- | --- | --- | --- | --- |
|  |  | **Mean_Measured_ ± SD (ng/mL)** | **Accuracy (%)** | **Precision (%)** | **Mean_Measured_ ± SD (ng/mL)** | **Accuracy (%)** | **Precision (%)** |
| BPA | 250 | 220.3 ± 11.1 | 88.1 | 5.0 | 259.8 ± 19.4 | 103.9 | 7.5 |
|  | 375 | 421.7 ± 37.1 | 112.4 | 8.8 | 383.8 ± 16.2 | 102.4 | 4.2 |
|  | 0.9 | 1.0 ± 0.01 | 110.1 | 1.1 | 0.9 ± 0.02 | 100.4 | 1.9 |
| BPF | 250 | 267.8 ± 31.3 | 107.1 | 11.7 | 245.5 ± 12.9 | 98.2 | 5.3 |
|  | 375 | 365.4 ± 18.7 | 97.4 | 1.6 | 376.9 ± 15.9 | 100.5 | 4.2 |
|  | 0.9 | 0.9 ± 0.1 | 99.8 | 5.6 | 0.9 ± 0.01 | 100.7 | 0.8 |
| BPS | 250 | 275.9 ± 30.6 | 110.4 | 11.1 | 261.1 ± 12.7 | 104.4 | 4.9 |
|  | 375 | 415.0 ± 18.7 | 110.7 | 4.5 | 396.6 ± 16.3 | 105.8 | 4.1 |
|  | 0.9 | 1.0 ± 0.04 | 109.1 | 3.8 | 0.9 ± 0.03 | 101.0 | 3.0 |

**Table S3** Linearity, LOD, LLOQ, and ULOQ in artificial urine.

| **Analyte** | ***r*^2^** | **Equation (1/x Weighting)** | **LOD (ng/mL)** | **LLOQ (ng/mL)** | **ULOQ (ng/mL)** |
| --- | --- | --- | --- | --- | --- |
| BPA | 0.9985 | y = 0.0954x − 0.0275 | 0.1 | 0.3 | 500 |
| BPF | 0.9952 | y =0.0964x − 0.0236 | 0.1 | 0.3 | 500 |
| BPS | 0.9981 | y = 0.0824x − 0.0228 | 0.1 | 0.3 | 500 |

| Characteristics | **BPA** | | | | | | |  | **BPF** | | | | | | |  | **BPS** | | | | | | |  | **ΣBisphenols** | | | | | | | |
| --- | --- | --- | --- | --- | --- | --- | --- | --- | --- | --- | --- | --- | --- | --- | --- | --- | --- | --- | --- | --- | --- | --- | --- | --- | --- | --- | --- | --- | --- | --- | --- | --- |
|  | GM | Min | Selected percentiles | | | Max | *p* value^a^ |  | GM | Min | Selected percentiles | | | Max | *p* value^a^ |  | GM | Min | Selected percentiles | | | Max | *p* value^a^ |  | GM | Min | Selected percentiles | | | Max | *p* value^a^ |  |
|  |  |  | 25^th^ | 50^th^ | 75^th^ |  |  |  |  |  | 25^th^ | 50^th^ | 75^th^ |  |  |  |  |  | 25^th^ | 50^th^ | 75^th^ |  |  |  |  |  | 25^th^ | 50^th^ | 75^th^ |  |  |  |
| All sample | 6.63 | 0.33 | 4.63 | 7.19 | 11.15 | 20.69 |  |  | 7.58 | 1.69 | 5.29 | 7.69 | 11.52 | 21.57 |  |  | 1.84 | 0.33 | 1.23 | 1.95 | 2.85 | 5.59 |  |  | 0.08 | 0.02 | 0.05 | 0.08 | 0.12 | 0.20 |  |  |
| Adults | 7.73 | 2.11 | 5.47 | 7.96 | 11.62 | 20.69 | <0.001 |  | 7.79 | 1.69 | 5.48 | 7.89 | 11.61 | 21.57 | 0.092 |  | 1.86 | 0.41 | 1.28 | 1.96 | 2.79 | 5.59 | 0.464 |  | 0.08 | 0.02 | 0.06 | 0.08 | 0.12 | 0.20 | <0.001 |  |
| Minors | 4.27 | 0.33 | 2.58 | 4.83 | 7.66 | 18.90 |  |  | 7.02 | 1.72 | 4.70 | 6.89 | 11.07 | 19.59 |  |  | 1.77 | 0.33 | 1.17 | 1.80 | 2.96 | 5.42 |  |  | 0.07 | 0.02 | 0.05 | 0.06 | 0.10 | 0.19 |  |  |
| Gender |  |  |  |  |  |  | 0.409 |  |  |  |  |  |  |  | 0.642 |  |  |  |  |  |  |  | 0.645 |  |  |  |  |  |  |  | 0.841 |  |
| Female | 6.84 | 0.33 | 4.66 | 7.44 | 11.57 | 20.69 |  |  | 7.50 | 2.07 | 5.33 | 7.68 | 11.38 | 21.57 |  |  | 1.85 | 0.33 | 1.16 | 1.98 | 2.96 | 5.42 |  |  | 0.08 | 0.02 | 0.06 | 0.08 | 0.12 | 0.18 |  |  |
| Male | 6.42 | 0.33 | 4.57 | 6.98 | 10.92 | 20.29 |  |  | 7.66 | 1.69 | 5.16 | 7.70 | 11.81 | 19.71 |  |  | 1.82 | 0.41 | 1.27 | 1.93 | 2.69 | 5.59 |  |  | 0.08 | 0.02 | 0.05 | 0.08 | 0.12 | 0.20 |  |  |
| Regional area |  |  |  |  |  |  | 0.357 |  |  |  |  |  |  |  | 0.108 |  |  |  |  |  |  |  | 0.707 |  |  |  |  |  |  |  | 0.175 |  |
| Northern | 6.82 | 0.33 | 5.17 | 7.66 | 11.20 | 20.29 |  |  | 7.55 | 1.80 | 5.13 | 7.82 | 11.52 | 19.59 |  |  | 1.83 | 0.33 | 1.27 | 1.97 | 2.79 | 5.26 |  |  | 0.08 | 0.02 | 0.05 | 0.08 | 0.11 | 0.19 |  |  |
| Central | 5.94 | 0.68 | 4.53 | 6.35 | 10.13 | 20.69 |  |  | 6.93 | 2.31 | 4.30 | 6.26 | 12.59 | 21.57 |  |  | 1.70 | 0.44 | 1.20 | 1.70 | 2.53 | 4.86 |  |  | 0.07 | 0.02 | 0.05 | 0.07 | 0.12 | 0.18 |  |  |
| Southern | 6.49 | 0.33 | 4.63 | 7.25 | 10.17 | 20.27 |  |  | 7.13 | 1.69 | 5.29 | 7.30 | 10.36 | 21.19 |  |  | 1.83 | 0.42 | 1.20 | 1.92 | 2.86 | 5.35 |  |  | 0.07 | 0.02 | 0.06 | 0.08 | 0.11 | 0.20 |  |  |
| Eastern | 6.40 | 0.53 | 4.12 | 6.63 | 11.70 | 19.58 |  |  | 8.25 | 3.10 | 5.64 | 7.99 | 12.71 | 19.71 |  |  | 1.96 | 0.53 | 1.17 | 2.07 | 3.14 | 4.83 |  |  | 0.08 | 0.03 | 0.05 | 0.09 | 0.12 | 0.20 |  |  |
| Remote island | 7.72 | 1.88 | 4.96 | 7.90 | 13.29 | 19.18 |  |  | 8.85 | 2.66 | 6.16 | 9.26 | 13.71 | 19.01 |  |  | 1.91 | 0.41 | 1.52 | 1.99 | 2.90 | 5.59 |  |  | 0.09 | 0.03 | 0.06 | 0.10 | 0.12 | 0.18 |  |  |
| **Adults** |  |  |  |  |  |  |  |  |  |  |  |  |  |  |  |  |  |  |  |  |  |  |  |  |  |  |  |  |  |  |  |  |
| Gender |  |  |  |  |  |  | 0.452 |  |  |  |  |  |  |  | 0.320 |  |  |  |  |  |  |  | 0.750 |  |  |  |  |  |  |  | 0.333 |  |
| Male | 7.96 | 2.28 | 5.75 | 8.02 | 11.81 | 20.29 |  |  | 8.05 | 1.69 | 5.54 | 8.10 | 11.86 | 19.71 |  |  | 1.89 | 0.41 | 1.40 | 1.97 | 2.76 | 5.59 |  |  | 0.09 | 0.02 | 0.06 | 0.09 | 0.13 | 0.20 |  |  |
| Female | 7.53 | 2.11 | 5.12 | 7.75 | 11.49 | 20.69 |  |  | 7.57 | 2.07 | 5.48 | 7.81 | 11.38 | 21.57 |  |  | 1.83 | 0.42 | 1.16 | 1.94 | 2.79 | 5.35 |  |  | 0.08 | 0.03 | 0.06 | 0.08 | 0.12 | 0.18 |  |  |
| Age (years) |  |  |  |  |  |  | 0.948 |  |  |  |  |  |  |  | 0.563 |  |  |  |  |  |  |  |  |  |  |  |  |  |  |  |  |  |
| 18–40 | 7.75 | 2.11 | 6.22 | 7.95 | 11.34 | 19.43 |  |  | 8.27 | 2.45 | 5.81 | 8.13 | 13.21 | 21.57 |  |  |  |  |  |  |  |  | 0.569 |  |  |  |  |  |  |  | 0.833 |  |
| 40–65 | 7.62 | 2.15 | 5.49 | 7.99 | 11.15 | 20.69 |  |  | 7.79 | 1.69 | 5.60 | 7.89 | 11.50 | 21.19 |  |  | 1.97 | 0.44 | 1.38 | 2.09 | 3.13 | 5.35 |  |  | 0.09 | 0.03 | 0.06 | 0.09 | 0.13 | 0.20 |  |  |
| 65 and older | 7.89 | 2.90 | 5.12 | 7.81 | 12.10 | 20.29 |  |  | 7.43 | 2.11 | 5.19 | 7.70 | 11.27 | 19.24 |  |  | 1.82 | 0.42 | 1.27 | 1.94 | 2.71 | 5.59 |  |  | 0.08 | 0.02 | 0.06 | 0.08 | 0.12 | 0.18 |  |  |
| **Minors** |  |  |  |  |  |  |  |  |  |  |  |  |  |  |  |  |  |  |  |  |  |  |  |  |  |  |  |  |  |  |  |  |
| Gender |  |  |  |  |  |  | 0.165 |  |  |  |  |  |  |  | 0.643 |  |  |  |  |  |  |  | 0.167 |  |  |  |  |  |  |  | 0.241 |  |
| Boy | 3.89 | 0.33 | 2.21 | 4.65 | 6.93 | 17.11 |  |  | 6.83 | 1.72 | 4.64 | 6.89 | 11.07 | 19.59 |  |  | 1.67 | 0.33 | 1.31 | 2.07 | 3.11 | 5.42 |  |  | 0.06 | 0.02 | 0.05 | 0.06 | 0.09 | 0.19 |  |  |
| Girl | 4.85 | 0.33 | 2.75 | 5.34 | 11.66 | 18.9 |  |  | 7.28 | 2.86 | 4.90 | 6.89 | 11.23 | 16.82 |  |  | 1.92 | 0.42 | 1.13 | 1.60 | 2.55 | 4.31 |  |  | 0.07 | 0.02 | 0.05 | 0.07 | 0.12 | 0.18 |  |  |
| Age (years) |  |  |  |  |  |  | 0.605 |  |  |  |  |  |  |  | 0.292 |  |  |  |  |  |  |  | 0.917 |  |  |  |  |  |  |  | 0.763 |  |
| 7–12 | 3.78 | 0.33 | 1.88 | 4.83 | 7.66 | 18.55 |  |  | 7.45 | 2.82 | 5.41 | 7.22 | 11.07 | 16.82 |  |  | 1.77 | 0.58 | 1.23 | 1.75 | 2.96 | 4.22 |  |  | 0.07 | 0.02 | 0.05 | 0.06 | 0.10 | 0.18 |  |  |
| 12–18 | 4.86 | 0.68 | 3.17 | 4.83 | 7.10 | 18.90 |  |  | 6.59 | 1.72 | 4.40 | 5.83 | 10.40 | 19.59 |  |  | 1.77 | 0.33 | 1.16 | 2.00 | 2.96 | 5.42 |  |  | 0.06 | 0.02 | 0.04 | 0.06 | 0.09 | 0.19 |  |  |

**Table S4** Distribution of BPs concentration (ug/L) in a sample of the general Taiwanese population (N = 366) (creatinine-unadjusted)

^a^ Comparison of urine creatinine-adjusted bisphenols levels between adult and minor (e.g. sex) using Mann–Whitney U test, above of two groups were using Kruskal- Wallis test, bold: *p* < 0.05.

| Characteristics | Item | Minors (<18 years, n = 95) | | | *p* value ^b^ |  | Adults (≥18 years, n = 271) | | | *p* value ^b^ |
| --- | --- | --- | --- | --- | --- | --- | --- | --- | --- | --- |
|  |  | All | Boy | Girls |  |  | All | Male | Female |  |
|  |  | n (%) | n (%) | n (%) |  |  | n (%) | n (%) | n (%) |  |
| **PCPs** ^a^ |  |  |  |  |  |  |  |  |  |  |
| Cumulative number of PCPs use | Low usage | 56 (66.7) | 36 (75) | 20 (55.6) | 0.061 |  | 107 (53.5) | 60 (72.3) | 47 (40.2) | **<0.001** |
|  | High usage | 28 (33.3) | 12 (25) | 16 (44.4) |  |  | 93 (46.5) | 23 (27.7) | 70 (59.8) |  |
| Body wash | No use/ Less than once a month | 2 (2.4) | 0 (0) | 2 (5.56) | 0.181 |  | 54 (26.7) | 14 (16.7) | 40 (33.9) | **0.006** |
|  | Once a month and more | 82 (97.6) | 48 (100) | 34 (94.4) |  |  | 148 (73.3) | 70 (83.3) | 78 (66.1) |  |
| Lotion | No use/ Less than once a month | 57 (67.9) | 36 (75) | 21 (58.3) | 0.106 |  | 72 (35.6) | 52 (62.7) | 20 (16.8) | **<0.001** |
|  | Once a month and more | 27 (32.1) | 12 (44.4) | 15 (41.7) |  |  | 130 (64.4) | 31 (37.3) | 99 (83.2) |  |
| Perfume | No use/ Less than once a month | 84 (100) | 48 (100) | 36 (100) | - |  | 173 (86.5) | 76 (91.6) | 97 (82.9) | 0.077 |
|  | Once a month and more | 0 (0) | 0 (0) | 0 (0) |  |  | 27 (13.5) | 7 (8.4) | 20 (17.1) |  |
| Nail polishes | No use/ Less than once a month | 80 (95.24) | 48 (100) | 32 (88.9) | **0.018** |  | 182 (91) | 83 (100) | 99 (84.6) | **<0.001** |
|  | Once a month and more | 4 (4.8) | 0 (0) | 4 (11.1) |  |  | 18 (9) | 0 (0) | 18 (15.4) |  |
| **Food** ^a^ |  |  |  |  |  |  |  |  |  |  |
| Fried food | No use/ Less than once a month | 8 (9.4) | 1 (2) | 7 (20) | **0.007** |  | 34 (19.2) | 13 (15.3) | 21 (22.8) | 0.203 |
|  | Once a month and more | 77 (90.6) | 49 (98) | 28 (80) |  |  | 143(80.8) | 72 (84.7) | 71 (77.2) |  |
| Barbecue food | No use/ Less than once a month | 9 (10.7) | 5 (10.4) | 4 (11.1) | 0.274 |  | 17 (8.5) | 12 (12.6) | 5 (4.8) | **0.048** |
|  | Once a month and more | 75 (82.3) | 43 (89.6) | 32 (88.9) |  |  | 182 (91.5) | 83 (87.4) | 99 (95.2) |  |
| Medication taken over the long term | Yes/No | 13/82  (13.7/86.3) | 9/46 (16.4/83.6) | 4/36 (10/90) | 0.373 |  | 110/161 (59.4/40.6) | 52/76  (40.6/59.4) | 58/85  (40.6/59.4) | 0.991 |

**Table S5** Personal care products (PCPs) use, food use and medication use in the study population

^a^ The missing values because of some participants did not answer this question

^b^ Comparisons for categorical variables were conducted using chi-square tests or fisher’s exact test , bold: *p* < 0.05.

**Table S6** Distribution of BPs concentration (μg/g Cr) in a sample of the general Taiwanese population with personal care products (PCPs) use, food use and medication use (N =366)

| Item | N | (%) | BPA | | |  | BPF | | |  | BPS | | |  | ΣBisphenols | | | |
| --- | --- | --- | --- | --- | --- | --- | --- | --- | --- | --- | --- | --- | --- | --- | --- | --- | --- | --- |
|  |  |  | Median | (25^th^-75^th^) | *p* value ^b^ |  | Median | (25^th^-75^th^) | *p* value ^b^ |  | Median | (25^th^-75^th^) | *p* value ^b^ |  | Median | (25^th^-75^th^) | *p* value ^b^ |  |
| **PCPs** |  |  |  |  |  |  |  |  |  |  |  |  |  |  |  |  |  |  |
| **PCPs usage ^a^** |  |  |  |  | 0.807 |  |  |  | 0.872 |  |  |  | 0.658 |  |  |  | 0.874 |  |
| No | 80 | 22.2 | 8.31 | (3.89, 15.90) |  |  | 7.90 | (4.59, 15.83) |  |  | 2.17 | (0.99, 4.11) |  |  | 0.09 | (0.05, 0.17) |  |  |
| Yes | 280 | 77.8 | 8.34 | (4.08, 16.38) |  |  | 8.79 | (5.03, 16.27) |  |  | 2.33 | (1.28, 4.06) |  |  | 0.09 | (0.05, 0.17) |  |  |
| **Cumulative number of PCPs use ^a^** |  |  |  |  | 0.872 |  |  |  | 0.454 |  |  |  | 0.881 |  |  |  | 0.697 |  |
| Low usage | 245 | 66.9 | 8.22 | (3.88, 16.33) |  |  | 8.80 | (4.54, 16.44) |  |  | 2.33 | (1.14, 4.08) |  |  | 0.09 | (0.05, 0.17) |  |  |
| High usage | 121 | 33.1 | 8.15 | (4.35, 15.16) |  |  | 8.63 | (6.05, 15.49) |  |  | 2.18 | (1.29, 3.86) |  |  | 0.09 | (0.06, 0.17) |  |  |
| **Body Wash ^a^** |  |  |  |  | **<0.001** |  |  |  | **<0.001** |  |  |  | **<0.001** |  |  |  | **<0.001** |  |
| No use/ Less than once a month | 56 | 19.6 | 16.10 | (8.74, 24.32) |  |  | 16.52 | (7.93, 25.79) |  |  | 3.55 | (2.18, 5.09) |  |  | 0.17 | (0.08, 0.27) |  |  |
| Once a month and more | 230 | 80.4 | 6.92 | (3.70, 13.64) |  |  | 7.83 | (4.42, 14.08) |  |  | 2.10 | (1.11, 3.54) |  |  | 0.08 | (0.04, 0.15) |  |  |
| **Lotion ^a^** |  |  |  |  | **<0.001** |  |  |  | **<0.001** |  |  |  | **<0.001** |  |  |  | **<0.001** |  |
| No use/ Less than once a month | 129 | 45.1 | 6.61 | (3.42, 12.80) |  |  | 7.02 | (3.85, 11.30) |  |  | 1.89 | (1.07, 3.26) |  |  | 0.08 | (0.04, 0.12) |  |  |
| Once a month and more | 157 | 54.9 | 9.60 | (5.12, 19.30) |  |  | 10.36 | (6.50, 19.83) |  |  | 2.59 | (1.51, 4.60) |  |  | 0.10 | (0.06, 0.20) |  |  |
| **Perfume ^a^** |  |  |  |  | 0.065 |  |  |  | 0.163 |  |  |  | 0.274 |  |  |  | 0.098 |  |
| No use/ Less than once a month | 257 | 70.2 | 7.63 | (3.97, 15.89) |  |  | 8.45 | (4.88, 15.57) |  |  | 2.18 | (1.25, 3.86) |  |  | 0.09 | (0.05, 0.16) |  |  |
| Once a month and more | 27 | 7.4 | 10.70 | (7.70, 16.95) |  |  | 11.31 | (6.69, 21.19) |  |  | 2.48 | (1.51, 5.09) |  |  | 0.11 | (0.08, 0.23) |  |  |
| **Nail polishes ^a^** |  |  |  |  | 0.434 |  |  |  | 0.872 |  |  |  | 0.618 |  |  |  | 0.639 |  |
| No use/ Less than once a month | 262 | 71.6 | 7.99 | (3.94, 16.12) |  |  | 8.79 | (4.73, 16.22) |  |  | 2.32 | (1.25, 3.94) |  |  | 0.09 | (0.05, 0.17) |  |  |
| Once a month and more | 22 | 6 | 9.54 | (6.48, 12.06) |  |  | 7.84 | (6.69, 15.00) |  |  | 2.25 | (1.56, 3.26) |  |  | 0.09 | (0.07, 0.13) |  |  |
| **Food** |  |  |  |  |  |  |  |  |  |  |  |  |  |  |  |  |  |  |
| **Fried food ^a^** |  |  |  |  | 0.142 |  |  |  | 0.463 |  |  |  | 0.303 |  |  |  | 0.304 |  |
| No use/ Less than once a month | 42 | 16 | 8.04 | (4.39, 15.96) |  |  | 7.90 | (5.92, 11.31) |  |  | 1.93 | (1.42, 3.78) |  |  | 0.08 | (0.05, 0.14) |  |  |
| Once a month and more | 220 | 84 | 6.68 | (3.40, 12.98) |  |  | 7.68 | (4.04, 14.26) |  |  | 1.91 | (1.00, 3.28) |  |  | 0.08 | (0.04, 0.14) |  |  |
| **Barbecue food ^a^** |  |  |  |  | 0.959 |  |  |  | 0.929 |  |  |  | 0.911 |  |  |  | 0.979 |  |
| No use/ Less than once a month | 26 | 9.2 | 8.24 | (3.47, 15.27) |  |  | 8.08 | (5.58, 12.90) |  |  | 2.60 | (0.71, 4.09) |  |  | 0.09 | (0.05, 0.15) |  |  |
| Once a month and more | 257 | 90.8 | 7.93 | (3.95, 15.06) |  |  | 8.12 | (4.47, 15.38) |  |  | 2.10 | (1.17, 3.69) |  |  | 0.09 | (0.05, 0.16) |  |  |
| **Long term use of medication ^a^** |  |  |  |  | **<0.001** |  |  |  | **<0.001** |  |  |  | **<0.001** |  |  |  | **<0.001** |  |
| No | 243 | 66.4 | 6.72 | (3.68, 13.64) |  |  | 7.48 | (4.27, 14.26) |  |  | 1.93 | (1.09, 3.59) |  |  | 0.08 | (0.04, 0.15) |  |  |
| Yes | 123 | 33.6 | 11.27 | (6.61, 21.62) |  |  | 10.47 | (6.92, 24.28) |  |  | 3.11 | (1.51, 4.89) |  |  | 0.11 | (0.08, 0.23) |  |  |

^a^ The missing values because of some participants did not answer this question

^b^ Comparisons for continue variables between two groups were conducted using Mann-Whitney U test, bold: *p* < 0.05.

Table S7 Distribution of BPs concentration (μg/g Cr) with different BMI weight status categories in the general Taiwanese population (N =366)

| Item | N | (%) | BPA | |  | BPF | |  | BPS | |  | ΣBisphenols | |
| --- | --- | --- | --- | --- | --- | --- | --- | --- | --- | --- | --- | --- | --- |
|  |  |  |  |  |  |  |  |  |  |  |  |  |  |
|  |  |  | Median (25th-75th) | *p* value ^b^ |  | Median (25th-75th) | *p* value ^b^ |  | Median (25th-75th) | *p* value ^b^ |  | Median (25th-75th) | *p* value ^b^ |
| Adults |  |  |  |  |  |  |  |  |  |  |  |  |  |
| BMI |  |  |  | 0.346 |  |  | 0.555 |  |  | 0.403 |  |  | 0.456 |
| Underweight | 13 | 4.8 | 8.05 (4.35-13.81) |  |  | 9.04 (4.54-22.90) |  |  | 2.02 (1.28-4.19) |  |  | 0.09 (0.06-0.20) |  |
| Healthy Weight | 140 | 51.7 | 10.71 (5.90-20.06) | |  | 9.96 (5.72-19.59) |  |  | 2.71 (1.46-4.90) |  |  | 0.11 (0.06-0.21) |  |
| Overweight | 118 | 43.5 | 8.82 (5.52-16.12) |  |  | 8.91 (5.58-15.49) |  |  | 2.24 (1.28-4.07) |  |  | 0.10 (0.06-0.16) |  |
| Minors |  |  |  |  |  |  |  |  |  |  |  |  |  |
| BMI |  |  |  | 0.889 |  |  | 0.576 |  |  | 0.332 |  |  | 0.747 |
| Underweight | 48 | 50.5 | 4.28 (1.57, 12.21) |  |  | 6.98 (3.94, 12.00) | |  | 2.15 (1.09, 3.31) | |  | 0.07 (0.04, 0.11) | |
| Healthy Weight | 34 | 35.8 | 4.07 (2.27, 6.78) |  |  | 6.38 (2.78, 12.04) | |  | 1.36 (0.92, 2.84) | |  | 0.06 (0.03, 0.10) | |
| Overweight | 13 | 13.7 | 3.97 (3.33, 8.65) |  |  | 5.70 (3.85, 10.21) | |  | 1.25 (0.99, 1.95) | |  | 0.05 (0.04, 0.09) | |

^a^ The defined of BMI weight status categories were based on Centers for Disease Control and Prevention (https://www.cdc.gov/healthyweight/assessing/bmi/adult_bmi/index.html)

^b^ Comparisons for continue variables between three groups were conducted using Kruskal-Wallis test, bold: *p* < 0.05.

**Table S8** Bisphenol daily intake (DI, ng /kg/day), hazard quotient (HQ) and hazard index (HI) in adults

|  | Adults (n= 271) | | | |  | Male (n=128) ^e^ | | | |  | Female (n=143) ^e^ | | | | |
| --- | --- | --- | --- | --- | --- | --- | --- | --- | --- | --- | --- | --- | --- | --- | --- |
|  | BPA | BPF | BPS | HI^a^ |  | BPA | BPF | BPS | HI^a^ |  | BPA | BPF | BPS | HI^a^ |  |
| DI - P50 | 2.29 | 2.35 | 0.58 | – |  | 2.06 | 1.96 | 0.49 | – |  | 2.72 | 2.54 | 0.69 | – |  |
| DI - P95 | 8.60 | 8.47 | 1.95 | – |  | 7.23 | 9.17 | 1.73 | – |  | 9.67 | 7.93 | 2.13 | – |  |
| DI - MAX | 18.36 | 18.25 | 4.17 | – |  | 18.36 | 18.25 | 4.17 | – |  | 17.23 | 13.47 | 3.42 | – |  |
| **Scenario 1** ^b^: Based on TDI by EFSA (2015) and thresholds derived by Mok et al (2021), Lin et al (2022) | | | | | | | | | | | | | |  |  |
| HQ/HI – P50 | 5.72×10^-4^ | 5.88×10^-4^ | 1.31×10^-4^ | 1.29×10^-3^ |  | 5.14×10^-4^ | 4.89×10^-4^ | 1.11×10^-4^ | 1.15×10^-3^ |  | 6.82×10^-4^ | 6.34×10^-4^ | 1.57×10^-4^ | 1.44×10^-3^ |  |
| HQ/HI – P95 | 2.15×10^-3^ | 2.12×10^-3^ | 4.44×10^-4^ | 4.55×10^-3^ |  | 1.81×10^-3^ | 1.66×10^-3^ | 3.94×10^-4^ | 4.70×10^-3^ |  | 2.42×10^-3^ | 1.98×10^-3^ | 4.84×10^-4^ | 4.51×10^-3^ |  |
| HQ/HI - MAX | 4.59×10^-3^ | 4.56×10^-3^ | 9.48×10^-4^ | 8.39×10^-3^ |  | 4.59×10^-3^ | 4.56×10^-3^ | 9.48×10^-4^ | 8.39×10^-3^ |  | 4.31×10^-3^ | 3.37×10^-3^ | 7.78×10^-4^ | 8.09×10^-3^ |  |
| **Scenario 2** ^c^: Based on TDI by EFSA (2023) and assumption that BPF and BPS have the same TDI | | | | | | | | | | | | | | |  |
| HQ/HI – P50 | 11.45 | 11.76 | 2.90 | 25.88 |  | 10.28 | 9.78 | 2.43 | 23.20 |  | 13.63 | 12.68 | 3.45 | 28.99 |  |
| HQ/HI – P95 | 43.02 | 42.37 | 9.77 | 92.23 |  | 36.16 | 45.86 | 8.66 | 94.80 |  | 48.35 | 39.63 | 10.64 | 91.22 |  |
| HQ/HI - MAX | 91.79 | 91.23 | 20.85 | 169.63 |  | 91.79 | 91.23 | 20.85 | 169.63 |  | 86.14 | 67.36 | 17.11 | 162.72 |  |
| **Scenario 3**: Based on RfD AA derived by Kortenkamp and Faust (2010) and thresholds derived by Mok et al. (2021) | | | | | | | | | | | | | |  |  |
| HQ/HI – P50 | 1.83×10^-4^ | – | 4.23×10^-5^ | 2.27×10^-4^ |  | 1.65×10^-4^ | – | 3.55×10^-5^ | 2.02×10^-4^ |  | 2.18×10^-4^ | – | 5.03×10^-5^ | 2.58×10^-4^ |  |
| HQ/HI – P95 | 6.88×10^-4^ | – | 1.43×10^-4^ | 8.69×10^-4^ |  | 5.78×10^-4^ | – | 1.26×10^-4^ | 7.45×10^-4^ |  | 7.74×10^-4^ | – | 1.55×10^-4^ | 9.65×10^-4^ |  |
| HQ/HI - MAX | 1.47×10^-3^ | – | 3.04×10^-4^ | 1.66×10^-3^ |  | 1.47×10^-3^ | – | 3.04×10^-4^ | 1.66×10^-3^ |  | 1.38×10^-3^ | – | 2.50×10^-4^ | 1.51×10^-3^ |  |
| **Scenario 4** ^d^**:** Based on RfD AA derived by Kortenkamp and Faust (2010), thresholds derived by Mok et al. (2021) and we assume the RfD AA of BPF were obtained by conversion to the same molar levels used for BPA. | | | | | | | | | | | | | | |  |
| HQ/HI – P50 | 1.83×10^-4^ | 2.14×10^-4^ | 4.23×10^-5^ | 4.35×10^-4^ |  | 1.65×10^-4^ | 1.78×10^-4^ | 3.55×10^-5^ | 3.85×10^-4^ |  | 2.18×10^-4^ | 2.30×10^-4^ | 5.03×10^-5^ | 5.01×10^-4^ |  |
| HQ/HI – P95 | 6.88×10^-4^ | 7.70×10^-4^ | 1.43×10^-4^ | 1.52×10^-3^ |  | 5.78×10^-4^ | 8.34×10^-4^ | 1.26×10^-4^ | 1.62×10^-3^ |  | 7.74×10^-4^ | 7.21×10^-4^ | 1.55×10^-4^ | 1.50×10^-3^ |  |
| HQ/HI - MAX | 1.47×10^-3^ | 1.66×10^-3^ | 3.04×10^-4^ | 2.85×10^-3^ |  | 1.47×10^-3^ | 1.66×10^-3^ | 3.04×10^-4^ | 2.85×10^-3^ |  | 1.38×10^-3^ | 1.22×10^-3^ | 2.50×10^-4^ | 2.74×10^-3^ |  |

^a^: HI =ΣHQ_BPs_

^b^: HQ =DI/TDI, BPA and BPF TDI= 4,000 ng/kg /day, BPS TDI= 4,400 ng/kg /day

^c^: HQ =DI/TDI, bisphenol A and its substitutes TDI= 0.2 ng/kg/day

^d^: HQ =DI/=RfD, BPA RfD = 12,500 ng/kg /day, BPF RfD = 11,000 ng/kg /day, BPS RfD = 13,700 ng/kg /day

^e^: Comparison of bisphenol A and its substitute’s DI between different sex using Mann-Whitney U test, the *p* value as follows: BPA (*p*= 0.063), BPF (*p*=0.066) and BPS (*p*=0.032).

**Table S9** Bisphenol daily intake DI (ng /kg/day), hazard quotient (HQ) and hazard index (HI) in minors

|  | Minors (n= 95) | | | |  | Boy (n=55) ^e^ | | | |  | Girl (n=40) ^e^ | | | |
| --- | --- | --- | --- | --- | --- | --- | --- | --- | --- | --- | --- | --- | --- | --- |
|  | BPA | BPF | BPS | HI^a^ |  | BPA | BPF | BPS | HI^a^ |  | BPA | BPF | BPS | HI^a^ |
| DI - P50 | 0.60 | 0.77 | 0.24 | – |  | 0.66 | 1.15 | 0.26 | – |  | 0.48 | 0.57 | 0.20 | – |
| DI - P95 | 3.68 | 5.48 | 1.38 | – |  | 4.63 | 9.80 | 2.13 | – |  | 2.61 | 2.84 | 0.55 | – |
| DI - MAX | 9.42 | 14.73 | 2.93 | – |  | 9.42 | 14.73 | 2.93 | – |  | 3.16 | 5.48 | 1.38 | – |
| **Scenario 1** ^b^: based on TDI by EFSA (2015) and thresholds derived by Mok et al (2021), Lin et al (2022) | | | | | | | | | | | | | |  |
| HQ/HI – P50 | 1.50×10^-4^ | 1.92×10^-4^ | 5.46×10^-5^ | 4.10×10^-4^ |  | 1.66×10^-4^ | 2.88×10^-4^ | 5.82×10^-5^ | 4.67×10^-4^ |  | 1.19×10^-4^ | 1.42×10^-4^ | 4.61×10^-5^ | 3.43×10^-4^ |
| HQ/HI – P95 | 9.20×10^-4^ | 1.37×10^-3^ | 3.13×10^-4^ | 2.23×10^-3^ |  | 1.16×10^-3^ | 2.45×10^-3^ | 4.84×10^-4^ | 4.25×10^-3^ |  | 6.52×10^-4^ | 7.10×10^-4^ | 1.25×10^-4^ | 1.46×10^-3^ |
| HQ/HI - MAX | 2.35×10^-3^ | 3.68×10^-3^ | 6.67×10^-4^ | 6.25×10^-3^ |  | 2.35×10^-3^ | 3.68×10^-3^ | 6.67×10^-4^ | 6.25×10^-3^ |  | 7.91×10^-4^ | 1.37×10^-3^ | 3.13×10^-4^ | 2.06×10^-3^ |
| **Scenario 2** ^c^: based on TDI by EFSA (2023) and assumption that BPF and BPS have the same TDI | | | | | | | | | | | | | | |
| HQ/HI – P50 | 2.99 | 3.85 | 1.20 | 8.30 |  | 3.32 | 5.75 | 1.28 | 9.44 |  | 2.38 | 2.83 | 1.02 | 6.99 |
| HQ/HI – P95 | 18.40 | 27.39 | 6.88 | 44.97 |  | 23.13 | 49.02 | 10.65 | 86.32 |  | 13.04 | 14.20 | 2.74 | 29.46 |
| HQ/HI - MAX | 47.08 | 73.65 | 14.67 | 126.22 |  | 47.08 | 73.65 | 14.67 | 126.22 |  | 15.81 | 27.39 | 6.88 | 41.80 |
| **Scenario 3**: based on RfD AA derived by Kortenkamp and Faust (2010) and thresholds derived by Mok et al. (2021) | | | | | | | | | | | | | |  |
| HQ/HI – P50 | 4.79×10^-5^ | – | 1.75×10^-5^ | 6.17×10^-5^ |  | 5.32×10^-5^ | – | 1.87×10^-5^ | 6.99×10^-5^ |  | 3.81×10^-5^ | – | 1.48×10^-5^ | 5.64×10^-5^ |
| HQ/HI – P95 | 2.94×10^-4^ | – | 1.00×10^-4^ | 3.74×10^-4^ |  | 3.70×10^-4^ | – | 1.55×10^-4^ | 5.26×10^-4^ |  | 2.09×10^-4^ | – | 4.00×10^-5^ | 2.38×10^-4^ |
| HQ/HI - MAX | 7.53×10^-4^ | – | 2.14×10^-4^ | 8.80×10^-4^ |  | 7.53×10^-4^ | – | 2.14×10^-4^ | 8.80×10^-4^ |  | 2.53×10^-4^ | – | 1.00×10^-4^ | 3.06×10^-4^ |
| **Scenario 4** ^d^**:** based on RfD AA derived by Kortenkamp and Faust (2010), thresholds derived by Mok et al. (2021) and we assume the RfD AA of BPF were obtained by conversion to the same molar levels used for BPA. | | | | | | | | | | | | | | |
| HQ/HI – P50 | 4.79×10^-5^ | 7.00×10^-5^ | 1.75×10^-5^ | 1.38×10^-4^ |  | 5.32×10^-5^ | 1.05×10^-4^ | 1.87×10^-5^ | 1.65×10^-4^ |  | 3.81×10^-5^ | 5.15×10^-5^ | 1.48×10^-5^ | 1.18×10^-4^ |
| HQ/HI – P95 | 2.94×10^-4^ | 4.98×10^-4^ | 1.00×10^-4^ | 7.61×10^-4^ |  | 3.70×10^-4^ | 8.91×10^-4^ | 1.55×10^-4^ | 1.48×10^-3^ |  | 2.09×10^-4^ | 2.58×10^-4^ | 4.00×10^-5^ | 4.94×10^-4^ |
| HQ/HI - MAX | 7.53×10^-4^ | 1.34×10^-3^ | 2.14×10^-4^ | 2.16×10^-3^ |  | 7.53×10^-4^ | 1.34×10^-3^ | 2.14×10^-4^ | 2.16×10^-3^ |  | 2.53×10^-4^ | 4.98×10^-4^ | 1.00×10^-4^ | 7.19×10^-4^ |

^a^: HI =ΣHQ_BPs_

^b^: HQ =DI/TDI, BPA and BPF TDI= 4,000 ng/kg /day, BPS TDI= 4,400 ng/kg /day

^c^: HQ =DI/TDI, bisphenol A and its substitutes TDI= 0.2 ng/kg/day

^d^: HQ =DI/=RfD, BPA RfD = 12,500 ng/kg /day, BPF RfD = 11,000 ng/kg /day, BPS RfD = 13,700 ng/kg /day

^e^: Comparison of bisphenol A and its substitute’s DI between different sex using Mann-Whitney U test, the *p* value as follows: BPA (*p*= 0.108), BPF (*p*= 0.005) and BPS (*p*= 0.026).

**References:**

Canada-Gazette. (2010). Part II 144 (21): 1806–1813. https://www.gazette.gc.ca/rp-pr/p2/2010/2010-10-13/pdf/g2-14421.pdf. Accessed 13 October 2010

Chen HC, Chang JW, Sun YC, Chang WT, Huang PC (2022) Determination of Parabens, Bisphenol A and Its Analogs, Triclosan, and Benzophenone-3 Levels in Human Urine by Isotope-Dilution-UPLC-MS/MS Method Followed by Supported Liquid Extraction. Toxics 10(1):21. https://doi.org/10.3390/toxics10010021

EFSA (European Food Safety Authority) (2015) Scientific opinion on the risks to public health related to the presence of bisphenol a (BPA) in foodstuffs. EFSA J 13(1): 3978. https://doi.org/10.2903/j.efsa.2015.3978

EFSA (European Food Safety Authority) (2023). Re-evaluation of the risks to public health related to the presence of bisphenol A (BPA) in foodstuffs. EFSA J. 21(4):6857. https://doi.org/10.2903/j.efsa.2023.6857

EMA (2011) Guideline on Bioanalytical Method Validation. European Union, London UK.

Health Canada (2015) Bisphenol A concentrations in Canadians, 2012 and 2013. https://www150.statcan.gc.ca/n1/pub/82-625-x/2015001/article/14208-eng.htm. Accessed 27 November 2015

Hurtado-Sánchez Mdel C, Acedo-Valenzuela MI, Durán-Merás I, Rodríguez-Cáceres MI (2015) Determination of chemotherapeutic drugs in human urine by capillary electrophoresis with UV and fluorimetric detection using solid-supported liquid-liquid extraction for sample clean-up. J Sep Sci 38(11):1990–1997. https://doi.org/10.1002/jssc.201401443

Kortenkamp A, Faust M (2010) Combined exposures to anti-androgenic chemicals: Steps towards cumulative risk assessment. Int J Androl 33(2):463–474. https://doi.org/10.1111/j.1365-2605.2009.01047.x

Lehmler HJ, Liu B, Gadogbe M, Bao W (2018) Exposure to bisphenol A, bisphenol F, and Bisphenol S in U.S. adults and children: The National Health and Nutrition Examination Survey 2013–2014. ACS Omega 3(6):6523–6532. https://doi.org/10.1021/acsomega.8b00824

Lin N, Ma D, Liu Z, Wang X, Ma L (2022) Migration of bisphenol A and its related compounds in canned seafood and dietary exposure estimation. Food Quality and Safety, 6:fyac006. https://doi.org/10.1093/fqsafe/fyac006

Liu H, Huang L, Chen Y, Guo L, Li L, Zhou H, Luan T (2015) Simultaneous determination of polycyclic musks in blood and urine by solid supported liquid–liquid extraction and gas chromatography–tandem mass spectrometry. J Chromatogr B Analyt Technol Biomed Life Sci 992:96–102. https://doi.org/10.1016/j.jchromb.2015.04.028

Marin, S.J. What’s the Best Way to do Supported Liquid Extraction? ISOLUTE® SLE+ User Guide Supported Liquid Extraction (2020). Available online: https://sampleprep.biotage.com/blog/whats-the-best-way-to-do-sle.

Matuszewski BK, Constanzer ML, Chavez-Eng CM (2003) Strategies for the Assessment of Matrix Effect in Quantitative Bioanalytical Methods Based on HPLC−MS/MS. Anal Chem 75:3019–3030. https://doi.org/10.1021/ac020361s

Mok S, Jeong Y, Park M, Kim S, Lee I, Park J, Kim S, Choi K, Moon HB (2021) Exposure to phthalates and bisphenol analogues among childbearing-aged women in Korea: Influencing factors and potential health risks. Chemosphere 264(Pt1):128425. https://doi.org/10.1016/j.chemosphere.2020.128425

Park C, Hwang M, Baek Y, Jung S, Lee Y, Paek D, Choi K (2019) Urinary phthalate metabolite and bisphenol A levels in the Korean adult population in association with sociodemographic and behavioral characteristics: Korean National Environmental Health Survey (KONEHS) 2012–2014. Int J Hyg Environ Health 222(5):903-910. https://doi.org/10.1016/j.ijheh.2019.02.003

Tschersich C, Murawski A, Schwedler G, Rucic E, Moos RK, Kasper-Sonnenberg M, Koch HM, Brüning T, Kolossa-Gehring M (2021) Bisphenol A and six other environmental phenols in urine of children and adolescents in Germany – human biomonitoring results of the German Environmental Survey 2014–2017 (geres V). Sci Total Environ 763:144615. https://doi.org/10.1016/j.scitotenv.2020.144615

Zhongzhe C, Jiang H (2019) Supported Liquid Extraction (SLE) in LC—MS Bioanalysis. John Wiley and Sons, NJ, USA, pp 76–84
